# Wanted and unwanted modifications of mRNA and their effect on gene expression and signaling

**DOI:** 10.1016/j.jbc.2026.113314

**Published:** 2026-07-06

**Authors:** Ankanahalli N. Nanjaraj Urs, Animesh Dali, Hani S. Zaher

**Affiliations:** Department of Biology, Washington University in St Louis, St Louis, Missouri, USA

**Keywords:** mRNA modification, RNA damage, translation, ribosome, the integrated stress response, ribosome quality control, ribotoxic stress signaling, collided ribosomes, translational control, gene expression

## Abstract

RNA plays essential roles in transmitting and decoding genetic information, as well as carrying out enzymatic functions during transcription and translation. The function of the polymer is determined by the unique structures of its four nucleobases, which dictate how the molecule interacts with itself, other RNAs, and proteins. As a result, modifications to the bases often impact RNA function by altering these interactions. While we have known for decades that tRNA, rRNA, and mRNA are modified, recent research has shed more light on the functional importance of these internal modifications in mRNAs. In addition to enzyme-mediated modifications, mRNA is also susceptible to damage-induced alterations. These arise from endogenous and exogenous alkylating, oxidizing, and cross-linking agents, as well as exposure to ultraviolet radiation. Many of these modifications severely disrupt tRNA–mRNA interactions, often leading to ribosome stalling. An integrating theme of this review is that both enzymatic and damage-induced modifications are actively sensed by the cell, with damage-induced modifications in particular engaging dedicated quality control pathways and stress–response programs that regulate gene expression and cellular fate. We begin this review by discussing the most abundant enzymatic mRNA modifications and focus on their impact on translation before turning to damage-induced lesions and the ribosome-based quality control machinery that resolves them. We conclude by discussing the remarkable discovery that damaged mRNA, through its impact on ribosome collisions, activates the integrated stress response and ribotoxic stress response to reprogram gene expression, and, under severe conditions, determine cell fate.

Nucleic acids play critical roles in storing, transmitting, and decoding genetic information. RNA performs additional catalytic roles during gene expression. These functions depend on the unique structures of the polymers endowed by the four nucleotide monomers that make them. In particular, the nitrogenous bases of the nucleotides are key in imparting the distinct identity assumed by the final polymer. Therefore, changes to the chemical structure of the bases could have profound effects on the function of DNA and RNA molecules. Indeed, the evolutionary choice of the four bases appears to have been driven largely by their relative chemical stability and inability to tautomerize at neutral pH. Yet, the presence of nucleophilic oxygen and nitrogen atoms within the bases allows them to react with multiple compounds. Some of these reactions are intentional and are driven by enzymes that cells utilize to regulate the function of DNA and/or RNA molecules (1). Other reactions are unintentional, resulting from nonenzymatic reactions, for example with chemical agents (2, 3). Some of these compounds, such as reactive oxygen species (ROS), are natural byproducts of cellular metabolism; whereas, others, such as ultraviolet (UV) light, are exogenous agents found in the environment. Regardless of the source of the chemical modification, even a small change to the nucleobase could dramatically alter the function of the molecule (4). mRNA’s function, during translation, for example, depends on its ability to base pair with tRNA molecules through codon–anticodon interactions (5). Modifications often change the base-pairing properties of the bases, either by altering their pairing preference or inhibiting it altogether and as a result may have dramatic consequences on the protein product encoded by the modified mRNA (6).

RNA molecules are structurally similar to DNA, having the very same basic chemical composition of four nitrogenous bases, covalently attached to a sugar-phosphate backbone. However, unlike DNA, RNA is primarily a single-stranded polymer, which makes it more exposed and accessible for reactive compounds and thus chemical damage. Indeed, alkylated and oxidized bases accumulate in RNA to much higher levels than in DNA (7, 8). Another major difference is the presence of ribose sugar in RNA in place of deoxyribose in DNA. The presence of a 2′-hydroxyl group on the sugar is a major factor contributing to the instability of RNA, leading to rapid hydrolysis of phosphodiester backbone, especially under alkaline conditions. This nature of RNA represents an important evolutionary advantage, enabling cells to quickly respond to environmental changes by rapidly synthesizing, utilizing, and degrading RNA molecules as needed, in contrast to DNA which is ∼200 times more stable at neutral pH and physiological levels of Mg^2+^ and is better suited for long-term genetic storage (9). Due to these differences, cells need not use the same strategies to deal with damage to DNA and RNA.

Given its paramount role in storing genetic information, organisms evolved exquisite mechanisms to maintain DNA’s integrity in response to environmental insults (10, 11, 12). As a result, cellular responses to DNA damage have been extensively studied for decades; these responses include repair processes and signaling pathways that regulate the cell cycle and fate. The consequences of RNA damage and cellular response to it, on the other hand, have not been appreciated until recently, presumably due to the long-held belief that the transient nature of the molecule did not warrant the evolution of dedicated quality control pathways for damaged RNA. This view was challenged with the discovery of RNA-surveillance pathways and the observation that damaged RNAs accumulate in disease states (13, 14). Indeed, inability to deal with RNA damage severely compromises cellular proteostasis through the production of aberrant protein products and sequestration of ribosomes (8, 15), and organisms appear to have evolved dedicated quality control pathways to quickly recognize and degrade damaged RNA. Notably, most cellular RNAs are also enzymatically modified, often producing modified bases that chemically resemble damaged ones (16, 17). Whether arising from enzymatic modifications or chemical damage, these alterations are actively sensed, and cells appear to have evolved dedicated systems to recognize and respond to them.

This review is mainly focused on the consequences and cellular responses to mRNA modifications and damage during translation. We begin by briefly summarizing key enzymatic modifications of the molecule, followed by a discussion of their effect on translation initiation, elongation, and termination. Our discussion then moves to nonenzymatic reactions that result in mRNA damage and how they impact the tRNA selection process during protein synthesis and how organisms utilize the ribosome to sense aberrant mRNAs to initiate their degradation. We conclude the review by summarizing exciting recent data showing that beyond activating quality control pathways, damaged mRNA through its action on ribosomes triggers the activation of stress–response pathways and can regulate cellular fate.

## RNA modifications

Soon after its discovery, it was realized that cellular RNA contains many nucleotides beyond the canonical four (adenosine, guanosine, cytidine, and uridine) (18, 19). Notably, over the past 2 decades, the list of these naturally modified nucleotides has gotten even longer owing to advances in mass-spectrometric and RNA-sequencing techniques. Currently, it is estimated that cellular RNA harbors close to 200 modified nucleotides (1, 20). Of these modifications, tRNA possesses the majority, followed by rRNA, and mRNA being a distant third. RNA molecules are modified at their 5′- and their 3′-ends and internally at their bases and sugar and phosphate backbone. The simplest modifications involve isomerization reactions, the addition of a methyl group and deamination of bases. More complex modifications involve the addition of multiple bulky groups, such as entire amino acids, at different atoms of the nucleobase and the ribose backbone (20). Modifications fulfill important roles, including critical functional ones, for tRNAs and rRNA. These include decoding, peptide-bond formation, and peptide release (21). They also alter the stability of the RNA molecule and its interaction with other RNAs and proteins (22). The role of these modifications in tRNA and rRNA functions is beyond the scope of this review, and the readers are encouraged to consult other reviews dedicated to these functional RNAs (23, 24, 25, 26, 27). Relevant to our discussion here, however, is the observation that newer, sensitive techniques have identified the presence of some of these modifications on mRNA (28, 29, 30, 31). In particular, we will discuss the potential importance of these modifications for the function of mRNAs and counterarguments suggesting that they are biological noise.

### mRNA capping

Following the initiation of synthesis of RNA transcripts in the nucleus, a series of essential trimming, modifications, and processing events occur. For mRNA, these modifications include 5′ capping, 3′ polyadenylation, and splicing. Upon completion of these modifications, the mature mRNA is exported into the cytoplasm, where it is either engaged by ribosomes for protein synthesis or targeted by RNA degradation machinery. These modifications are crucial in regulating mRNA stability, nuclear export, translation efficiency, gene expression, and proper functioning of the cellular process (32). Notably, all these modifications occur cotranscriptionally, taking advantage of the unique structure and regulation of RNA polymerase II (RNAP II) (33, 34).

**Figure 1:**
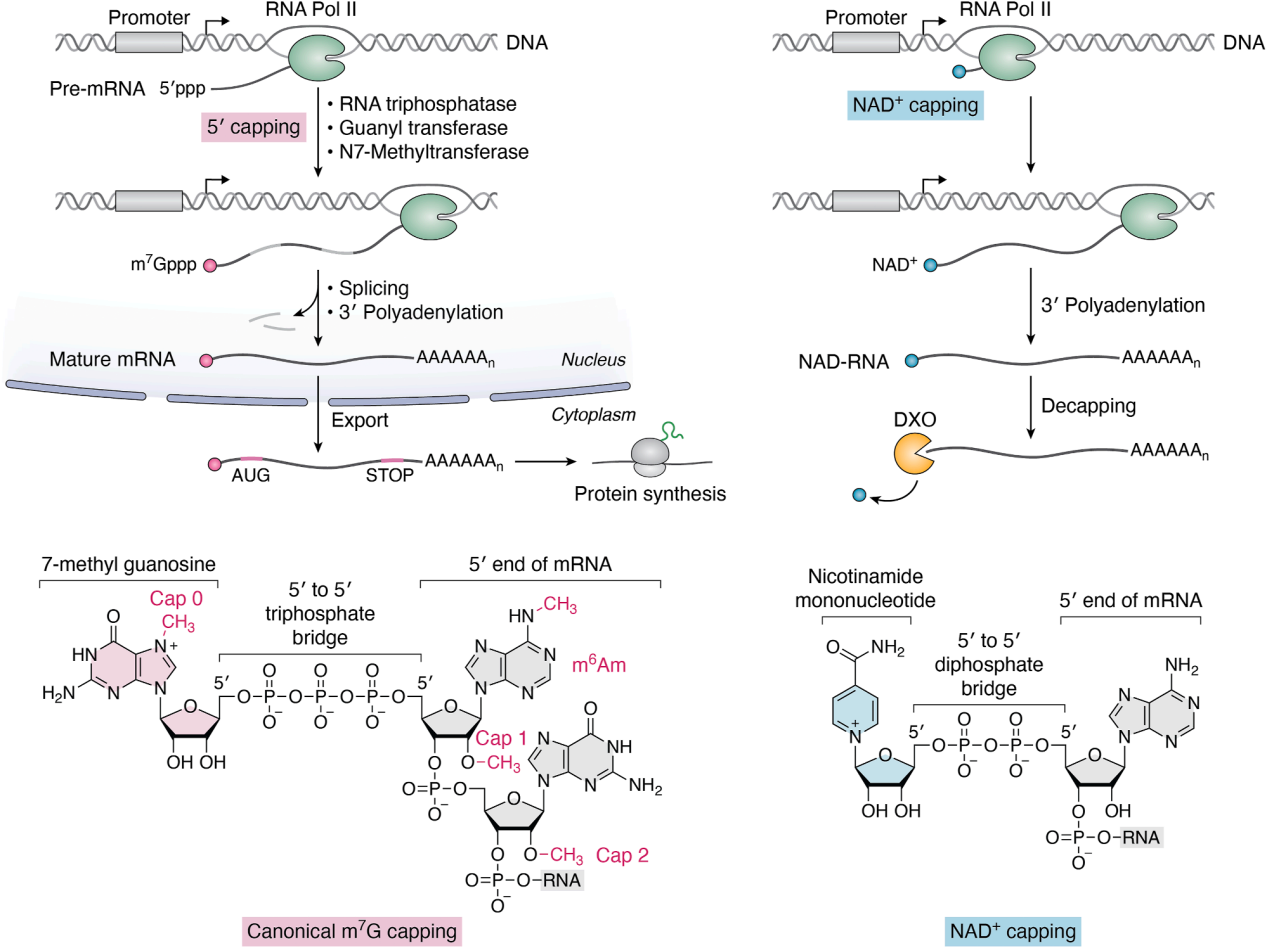
**mRNAs capping and its function.** The *top left panel* is a schematic showing the cotranscriptional addition of the canonical m7G cap structure. The *bottom left panel* shows the chemical structure of the modification. It consists of a 7-methylguanosine (m^7^G) linked to the first transcribed nucleotide *via* a 5′–5′ triphosphate bridge. Methylation at the N7 position of guanosine generates the cap-0 structure, while additional 2′-O-methylation of the first and second nucleotides produces the cap-1 and cap-2 structures, respectively. In addition, if 2′-O-methyl-adenosine is the first nucleotide after the m7G cap, it can undergo further methylation at its N6 position to form N6-2′-O-dimethyladenosine (m6Am) modification. The *top right panel* is a schematic showing the formation of NAD-RNA and its subsequent decapping by DXO. The *bottom right panel* depicts the chemical structure of the NAD-RNA, composed of a nicotinamide mononucleotide (NMN) connected to an AMP moiety *via* a 5′ to 5′ diphosphate bridge. The 3′ hydroxyl group of the AMP moiety of NAD connects to the RNA body.

Capping of the nascent pre-mRNA occurs soon after RNAP II starts transcription, having completed the synthesis of only 20 to 30 nucleotides (35, 36) (Fig. 1). The entire reaction requires at least three enzymatic reactions. It starts with an RNA triphosphatase, which removes that γ-phosphate from the 5′-triphosphate of the pre-mRNA. The second reaction, catalyzed by a guanyl transferase, involves the transfer of GMP (guanosine monophosphate) from GTP to the β-phosphate of 5′ mRNA. This step essentially caps the mRNA forming a 5′-5′ triphosphate linkage. The cap structure is then decorated by the addition of a methyl group on N7 of the terminal guanosine through the action of RNA methyl transferase (Table 1). This minimal cap structure, termed cap 0, is required for downstream processing of the mRNA through splicing and polyadenylation (37, 38, 39) (Fig. 1). This is accomplished through the recruitment of the nuclear cap-binding complex (CBC) composed of CBP20 and CBP80 (40). CBC promotes pre-mRNA splicing through interactions with the U4/U6.U5 tri-snRNP of the spliceosome (41). On the other hand, it contributes to the mRNA 3′-end formation and polyadenylation (42). Following processing, CBC-bound mRNAs are then exported to the cytoplasm, during which CBC plays a direct role through interactions with nuclear export factors including those of the transcription export complex (43, 44). In the cytoplasm, the cap structure is recognized by the initiation factor eIF4E of the eIF4F complex (Table 1). This step is required for mRNA activation and recruitment of the small ribosome subunit for canonical initiation to take place (45). At the same time, the cap structure renders the mRNA resistant to the exonucleolytic action of the 5′-3′ exonuclease (XRN1), which is unable to degrade the mRNA unless the cap structure is removed by the decapping enzyme (46, 47) or the mRNA is endonucleolytically cleaved.

**Table 1:** Proteins mediating cap structure and translation initiation

Category	Protein/complex	Role/function
Cap structure and cap-proximal modifiers	RNA triphosphatase	Removes the γ-phosphate from the 5′-triphosphate of nascent pre-mRNA
	Guanyl transferase	Transfers GMP to the β-phosphate of the 5′ end, forming the 5′-5′ triphosphate cap linkage
	N7-methyltransferase	Adds a methyl group to N7 of the terminal guanosine, producing the minimal cap-0 structure
	PCIF1	Methylates the N6 of the +1 adenosine to form m^6^Am; recruited co-transcriptionally by the Ser5-phosphorylated CTD of RNAP II
	CBC (CBP20/CBP80)	Nuclear cap-binding complex; promotes splicing, 3′-end formation/polyadenylation, and nuclear export
	TREX complex	Couples splicing to nuclear export; CBC-bound mRNAs are exported *via* this complex
	eIF4E/eIF4F complex	Recognizes the m7G cap and recruits the 43S PIC for canonical translation initiation
	DCP2	Decapping enzyme; removes the 5′ cap to initiate mRNA decay; inhibited by m6Am modification
	DXO	Removes noncanonical NAD^+^ caps (de-NADding); its absence leads to accumulation of NAD^+^-capped mRNAs
Translation initiation factors	eIF3	Integral component of the 43S PIC
	eIF4G	Scaffolding subunit of eIF4F; required for canonical cap-dependent initiation
	eIF5B	GTPase involved in 60S subunit joining during late translation initiation

As described above, the minimal cap 0 structure appears to be important for export, stabilization, and translation of the mRNA. However, the structure is typically further modified, especially at position +1 and +2 (Fig. 1). The most common modification is 2′-O methylation of the 5′ terminal nucleotide of the mRNA to form the so-called cap 1 structure, which has been documented to serve a role in distinguishing self from nonself mRNA. In particular, pattern recognition receptors RIG-I and MDA5 recognize viral RNAs and activate the downstream interferon response. Both of these recognize double-stranded RNA, but their binding is inhibited by the presence of the cap 1 structure (48, 49). More recently, it was shown that certain cap 1 containing mRNAs can be modified further when the transcript starts with an adenosine (50, 51, 52). In addition to 2′-O-methylation, the base of the terminal nucleotide can be methylated at N6 to form N6-2′-O-dimethyladenosine (m6Am) (Fig. 1). N6-methylation occurs cotranscriptionally through the recruitment of the methyl transferase PCIF1 to the initiating RNAP II by interacting with its Ser5-phosphorylated CTD (50, 51, 52, 53). The modification is reversible through the action of the oxidative demethylase FTO, suggesting that the methylation is dynamic and can be used to regulate the expression of the modified mRNA (54). Interestingly, the methylation inhibits DCP2-catalyzed decapping of mRNAs (Table 1). However, how N6-methyladenosine (m6A) methylation of the terminal adenosine impacts the function of the mRNA remains a subject of debate. One group argued that it stabilizes the transcript, based on correlation between the modification and RNA levels, and that it has little to no role during translation based on ribosome profiling data of PCIF1 KO cells (50). Yet, another group argued for the opposite, providing data that showed m7G-m6Am-capped reporter mRNAs transfected into PCIF1 KO MEL624 cells are translated less efficiently than m7G-Am-capped ones (52). At the same time, they argued that the stability of m6Am-modified mRNAs is unaffected in the absence of PCIF1. In mammals, translation and mRNA stability are somewhat correlated, which could potentially confound disentangling their effect on gene expression.

In addition to canonical cotranscriptional capping of mRNAs, some transcripts were shown to be capped by nicotinamide linked through a 5′-5′ diphosphate to a terminal adenosine (55, 56, 57) (Fig. 1). The addition of this cap structure appears to be enzymatically distinct from canonical capping as it does not require additional machinery to incorporate it. Instead, in the most likely scenario, during initiation of transcription of pre-mRNAs with terminal adenosine, RNAP II sometimes utilizes NAD^+^—a dinucleotide cap with adenosine at one terminus—to start transcription in place of ATP (58, 59). The role of NAD^+^ capping is currently not completely understood. The modification inhibits cap-dependent translation and is removed through the action of the decapping enzyme DXO (60) (Fig. 1). Given that NAD^+^ capping occurs at low levels with little to no features distinguishing those mRNAs from canonically capped ones (60), it is entirely plausible that this alternative mode of capping represents biological noise. More specifically, it is possible that NAD^+^ capping signifies an error during transcription. The concentration of NAD^+^ can be as high as 0.5 mM in mammalian cells (61), which is about an order of magnitude lower than that of ATP. It is well-documented that RNAPs from all domains of life can efficiently initiate transcription with cap analogs, so long as one of the two nucleotides in the cap structure is the terminal 1 of the RNA, a property of the polymerases exploited to generate capped mRNAs *in vitro* (62). Therefore, the observation that NAD^+^ capping occurs at a frequency of 1 to 5% (60) could reflect this property of the polymerase together with the relative high availability of the dinucleotide in the cell.

### N6-methyladenosine

**Figure 2:**
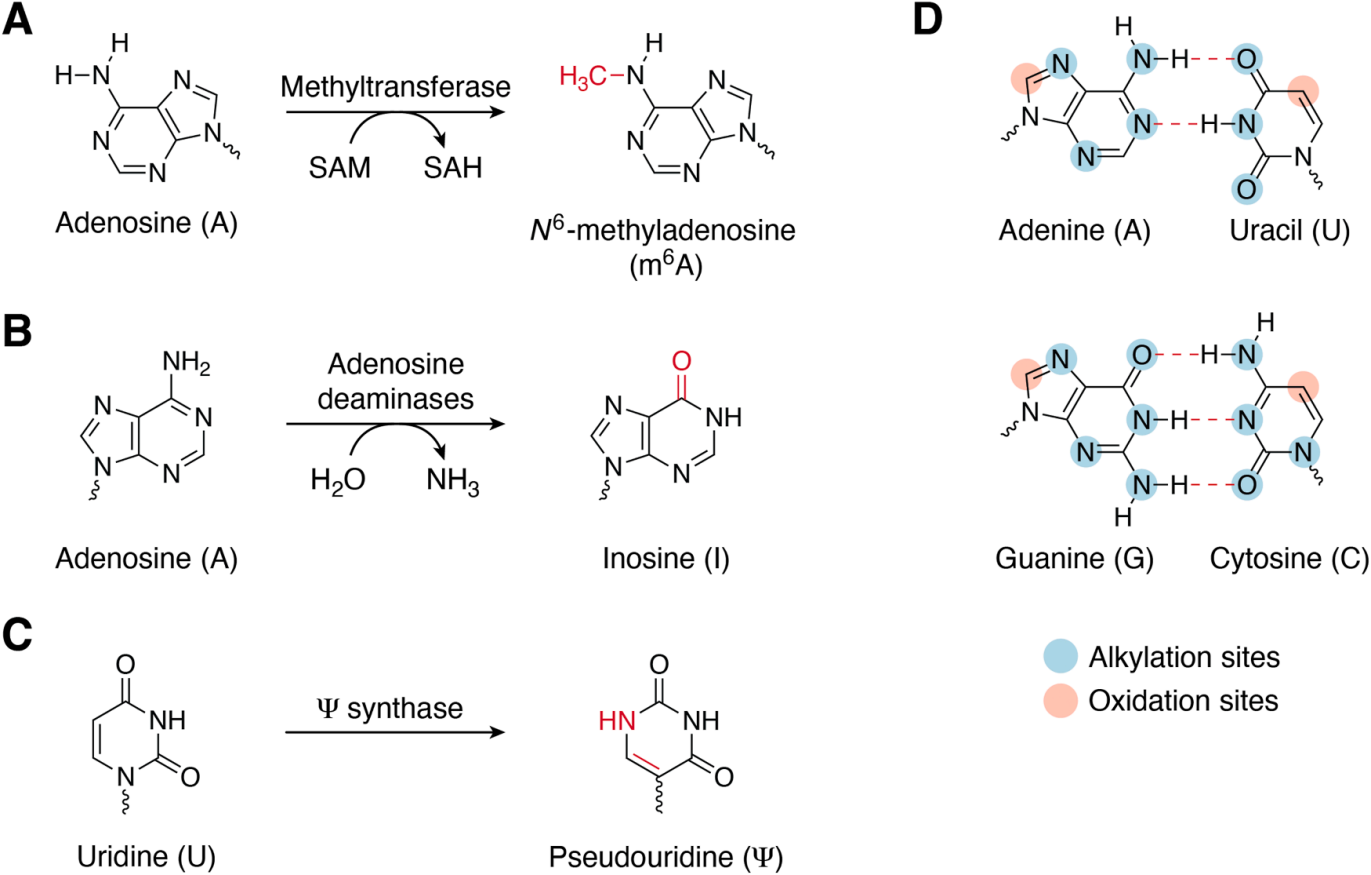
**Chemical structures of RNA, their interactions, and the most common internal modifications in mRNA.***A*, methylation of the N6-adenosine in mRNA as catalyzed by its dedicated methylase transferase complex that uses SAM as methyl donor. *B*, deamination of adenosine to inosine through a hydrolytic attack at C6 of the adenine base is catalyzed by adenosine deaminases acting on RNA enzymes. *C*, pseudouridine synthases catalyze the site-specific isomerization of uridine (U) to pseudouridine (Ψ) in RNA. *D*, canonical RNA base pairing is disrupted by alkylation and oxidation adducts. Modification sites are shown in *red* (oxidation) and *blue* (alkylation), respectively. Adapted from Yan *et al*. (3).

The m6A modification (Fig. 2*A*) is the most prevalent internal modification in mRNA comprising up to 1% of total adenosines with each mRNA harboring 3 to 4 modifications on average. Notably, m6A is one of few modifications to have a dedicated mRNA-specific machinery for depositing it (63, 64, 65, 66, 67, 68, 69). As far as we know, almost all other internal mRNA modifications (excluding deamination) repurpose tRNA and rRNA modifying enzymes. m6A modification of mRNA was discovered more than half a century ago using metabolic labeling of hepatoma cells with a radiolabeled methyl-donor precursor (70). Interestingly, unlike tRNA and rRNA, which contain a complex mixture of methylated nucleotides (on the base as well as the ribose backbone), mRNA was found to almost exclusively contain the one modification of m6A (71). The m6A modification was understudied for more than 3 decades apart from a few studies that showed the modification was present in mammalian cell lines and viral RNAs (72, 73, 74, 75). However, m6A has garnered much more attention over the past decade due to the discovery that m6A can be removed by the oxidative demethylases FTO and ALKBH5 (53, 76, 77) combined with the development of sequencing technologies to map m6A in RNAs (64, 76). The modification is deposited on the pre-mRNA cotranscriptionally by the methyl transferase complex, also known as the "writer" complex, comprising METTL3, METTL14, and WTAP (63, 65, 67) (Table 2). m6A sites are conserved and can regulate numerous facets of RNA metabolism through their interaction with "reader" proteins (64, 78, 79). In this review, we will only summarize its impact on translation.

**Table 2:** Key protein components of RNA modification machinery

Category	Protein/complex	Role/function
m6A modification machinery	METTL3–METTL14–WTAP ("writer" complex)	Deposits m6A cotranscriptionally on pre-mRNA at conserved RRACH motifs
	FTO	Oxidative demethylase that removes both internal m6A and cap-proximal m6Am
	ALKBH5	Oxidative demethylase that removes m6A; impacts RNA metabolism and fertility
	YTHDF1/2/3	Reader proteins that regulate translation, decay, or P-body storage of m6A-modified mRNAs
	YTHDC2	Reader/helicase that promotes translation of m6A-modified mRNAs in the CDS
	YTHDC1	Proposed nuclear reader for m6A-containing RNA; accelerates target mRNA degradation
Secondary nucleotide modifiers	ADAR1	Deaminates adenosine to inosine in dsRNA; ubiquitously expressed; suppresses antiviral response activation to endogenous RNA
	ADAR2	Deaminates adenosine to inosine in dsRNA; neuron-enriched; responsible for recoding transcripts including GluR-B (glutamate receptor)
	ADATs (adenosine deaminases acting on tRNAs)	Modify the wobble position (A34) of tRNA anticodons, expanding codon recognition
	PUS enzymes	Pseudouridine synthases; install pseudouridine (Ψ) at specific tRNA sites. PUS1 has been suggested to modify mRNAs as well.

Initial mapping studies found the modification to occur throughout the mRNA sequence but enriched in the 3′-UTR around the stop codon (64, 80). Nevertheless, the CDS region is modified and in principle can impact tRNA selection by the ribosome during elongation (81). Although methylation of N6 of adenosine does not alter its base-pairing preference, it slightly reduces the RNA duplex stability (82). As a result, the modification destabilizes codon–anticodon interaction, which is reflected by an overall reduced rate of peptide bond formation in a bacterial *in vitro* system, which slightly increases the error frequency (83, 84, 85). The overall reduction is context dependent and can vary from 3-fold to 40-fold. However, on ribosomes programmed with the canonical m6A RRACH sites, the decrease in peptide-bond formation is about 10-fold (86). In principle, this slowdown in peptide-bond formation may have evolved to regulate cotranslational events, such as protein folding, but a correlation between the location of the modification and protein interdomain regions is yet to be identified. More recently, it was shown that methylation of A triggers cotranslational mRNA decay, which is triggered by slowed decoding by the ribosome and was termed CDS-m6A decay (81). These observations appear inconsistent with previous data showing that although m6A in the CDS region slows ribosome decoding, it promotes translation through the “reader” protein YTHDC2 (87). These contradictory results by different groups are a common theme in the m6A field, presumably because of the context of the modification and the layered complexity associated with the modification. Furthermore, many of these interpretations are made by comparing cells in which the entire methyl transferase machinery is abrogated to cells that have the machinery present. This is a blunt approach, and many of the effects could be indirect. To the best of our knowledge, no study to date has studied an individual mRNA with m6A modification at a particular site and compared its translation ability and stability to an unmodified one in cells. Confounding some of these conclusions is also the observation that in the CDS regions, the frequency at which a particular site is modified is substoichiometric maxing at 20% (88). As a result, it is currently unclear why organisms would have evolved these sorts of mechanisms to regulate only a fraction of an mRNA transcript.

A more recent study has provided an additional model for how m6A in CDS initiates mRNA degradation through induction of ribosome collisions. Pronounced mRNA stalling based on the frequency of m6A in CDS leads to ribosome pileup on the transcript, eventually recruiting YTHDFs for m6A mRNA storage or degradation in P bodies (89). How YTHDF proteins recognize collided ribosomes is not completely understood. Importantly, the requirement for an additional mechanism to sense collided ribosomes is puzzling because there are other mechanisms known to trigger mRNA decay in response to ribosome stalling (15, 90, 91, 92, 93, 94). It is difficult to imagine how stalling on m6A would be distinct from that experienced by the ribosome on damaged mRNA as both are expected to result in delayed binding of aminoacylated tRNA to the A site.

**Figure 3:**
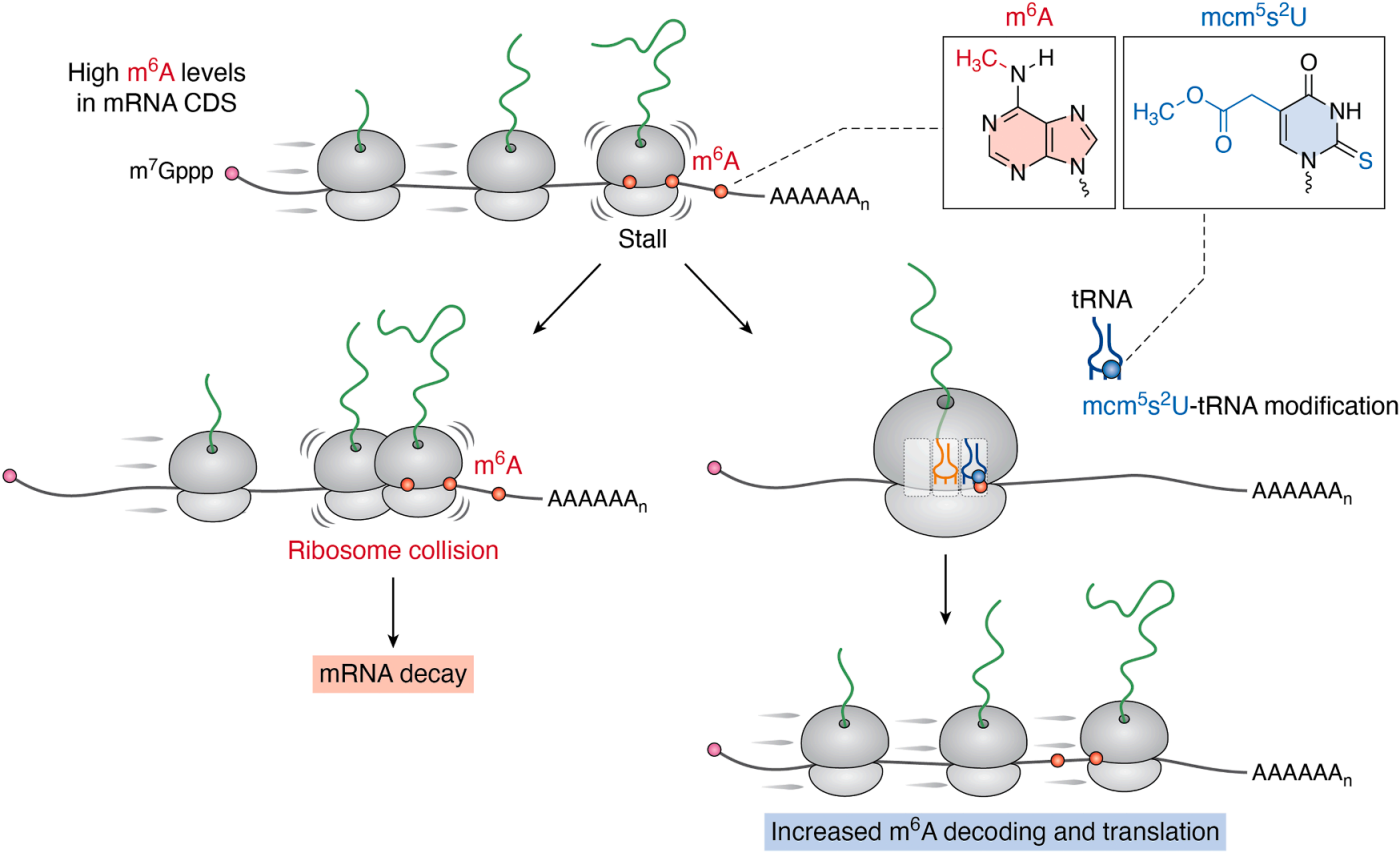
**Consecutive N6-methyladenosine (m6A) in mRNA CDS induces ribosome stalling and couples it to accelerated decay.** High levels of the m6A modification in the mRNA CDS slow down the ribosome significantly, resulting in stalling and potentially, ribosome collisions, which in turn trigger mRNA degradation. The 5-methoxycarbonylmethyl-2-thiouridine (mcm5s2U) modification in the tRNA anticodon loop enhances the decoding efficiency of m6A-containing codons, increasing the speed of translation of m6-modified mRNAs and thus stabilizing them.

Notably, at the same time a study by Linder *et al.* (95) identified an additional layer of RNA-modification regulation that links m6A-modified mRNAs to specific tRNA wobble-uridine modifications. The authors show that the mcm5s2U modification in tRNA enhances the decoding efficiency of codons that carry m6A on their corresponding mRNAs. This cooperative interaction mitigates the translational slowdown normally caused by m6A and, as a result, can reduce m6A-dependent mRNA degradation (Fig. 3). These findings suggest a mechanism by which certain m6A-modified transcripts may evade decay, particularly in proliferative cancer cells where tRNA modification pathways are often upregulated. By boosting decoding efficiency through mcm5s2U, cancer cells may selectively stabilize m6A marked mRNAs that support growth and survival.

As stated earlier, m6A is mainly found in the 3′-UTR of mRNAs, where it has been implicated in regulating translation through the action of reader proteins. Initial studies suggested that m6A promotes mRNA activation and translation through looping by binding YTHDF1 in the presence of eukaryotic initiation factors (eIFs) (96). Later studies argued that binding of m6A by YTHDF proteins does not alter translational efficiency, but instead it promotes mRNA degradation (89, 97) (Table 2). In agreement with these arguments, a more recent study reported a negative correlation between m6A levels and ribosome occupancy and suggested that the modification promotes their localization to the translationally repressed P bodies by binding the RNA-binding protein IGF2BPs (98).

In addition to the CDS and 3′-UTR, m6A is also found in the 5′-UTR of mRNAs, albeit to much lower levels than the other regions (80). In a provocative study, it was shown that a single m6A residing in the 5′-UTR can recruit ribosomes in the absence of the cap-binding initiation factor eIF4E (99). It was proposed that this cap-independent translation initiation relies on the ability of the initiation factor eIF3 to bind m6A directly and be recruited to the mRNA in the absence of eIF4G, which requires eIF4E to be present under normal conditions. Since eIF3 is an integral component of the ribosomal preinitiation complex (PIC), this model offered an internal ribosome entry site type of initiation. In support of this model, it was shown that in HeLa cells depleted of m6A through the knockdown of the methyltransferase METTL3, mRNAs harboring the modification in their 5′-UTR displayed lower translational efficiencies relative to ones having the modification elsewhere. This was in direct contrast to other studies showing no correlation between m6A in the 5′-UTR and translational efficiency (98). Instead, others suggested that METTL3 promotes translation initiation in the cytoplasm, independent of its nuclear methyltransferase function (100). Indeed, a more recent study failed to see any evidence for cap-independent translation in the presence of the modification in the 5′-UTR. In this study, the authors also took advantage of the observation that the modification increases in 5′-UTR under oxidative stress and evaluated available ribosome profiling data to assess the effect of increased modification on translation efficiency. Remarkably, the authors found the efficiencies to decrease similarly for all transcripts regardless of whether the 5′-UTR was annotated to contain the modification or not (101). *In vitro* single-molecule reconstitution experiments corroborated these findings, in which the modification was found to have no effect on the recruitment of the PIC, and late initiation stages of eIF5B binding and 60S subunit joining. Notably, m6A is typically enriched at -3′ position relative to the AUG start codon, and structural studies showed that this base interacts with eIF2α and not eIF3. Furthermore, while the methyl group can stack with R55 of the protein, the added energetics from these interactions are traded for the loss of interaction with ribosomal protein uS7 (101). As a result, it was concluded that the net effect of the methyl group on the interaction with the 43S PIC is negligible. These newer findings reiterate the need for further investigation into how m6A influences early translation initiation events.

In contrast to its effect on tRNA selection, much less is known about the impact of m6A on peptide release. Nevertheless, *in vitro*, the efficiency of release factor-catalyzed termination on stop codon has been shown to decrease by about 6-fold in the presence of the modification (85). Notably, the efficiency of termination on near-stop codons decreased by only 3-fold. These observations have been used to suggest that m6A reduces the fidelity of peptide release on the ribosome. However, given the modest (2-fold) decrease in accuracy, it is unclear whether the effects of m6A on release factor activity are physiologically relevant or not. More importantly, stop codons do not contain the canonical sequence motif recognized by the methyltransferase complex and as a result are not known to be modified to high levels. Therefore, it is highly unlikely that m6A regulates translation termination in cells.

### Deamination of adenosine to inosine

Initially discovered through their ability to edit double-stranded RNA, adenosine deaminases acting on RNA (ADARs) catalyze the hydrolytic attack on carbon 6 of the nucleotide, converting it to inosine (102, 103, 104, 105) (Fig. 2*B*). The deamination reaction alters the base-pairing preference of the nucleotide (102, 104). Indeed, the related adenosine deaminases acting on tRNAs (ADATs) modify the wobble position of the tRNA anticodon A34, expanding the repertoire of codons recognized by the modified tRNA (106). Of note, inosine forms a Watson–Crick base-pair geometry when paired with cytidine (105). Humans have two catalytically functional ADARs: ADAR1 is ubiquitously expressed and plays a critical role in innate immune response, suppressing the activation of the antiviral response to self RNA (107, 108, 109, 110, 111, 112, 113, 114); ADAR2 is expressed in the brain, where it plays a major role in recoding mRNAs, and in particular the glutamate receptor transcript (115, 116, 117, 118, 119) (Table 2). Until recently, it was thought that inosine is unambiguously decoded as guanosine, given its similarity to this nucleotide. However, more recent studies have suggested that similar to how the modification behaves on tRNAs, inosine can be decoded as uridine and adenosine depending on its sequence context and location within the codon (120). The structural basis for this altered base pairing remains unclear as of yet. Regardless and even though most editing occurs in noncoding RNA (Alu repetitive elements), introns, and 3′-UTRs, >1500 editing sites have been identified in the coding regions of human mRNA. Of note, however, the editing levels at these sites are low (average of 5%), with 40% of the sites residing in Alu-like elements (121). Therefore, the physiological role of the modifications on these sites remains to be revealed, especially in light of the observation that seizures and eventual lethality in ADAR2-knockout mice can be rescued by a single point mutation in the glutamate receptor transcript that “corrects” the editing target of the deaminase (122).

### tRNA and rRNA modifying enzymes deposit modifications on mRNA

In addition to 5′-capping, m6A modification, and adenosine deamination, recent advances have identified several modifications in the mRNAs from all domains of life (123). These modifications, however, do not employ mRNA-dedicated machinery but instead exploit enzymes that typically target rRNA and tRNA. Also, unlike the mRNA-specific modifications, these “pan-RNA” modifications are lowly abundant in mRNA. While pseudouridine can be deposited at a particular site within an mRNA to a relatively high frequency of 10 to 30%, the stoichiometry of the other modifications are typically less than 1 to 5% (123, 124, 125, 126, 127). As such, it is unclear to what extent these modifications are programmed by cells to alter gene expression *versus* being off targets of the tRNA–rRNA-modifying machinery. Nevertheless, given the immense interest in pseudouridine, we will briefly discuss this modification and its effect on mRNA translation.

Pseudouridine is the most abundant modification in total RNA, constituting ∼30% and ∼9% of total uridines in tRNA and rRNA, respectively (128, 129, 130, 131). The pseudouridylation reaction requires the isomerization of uridine to pseudouridine (Ψ), whereby the glycosidic bond between N1 of the base and C1′ in the sugar is cleaved and replaced with a C5-C1′ bond (130) (Fig. 2*C*). Therefore, the modification does not change the Watson–Crick base pairing preference of the base. Although the addition of an amino group to position 1 in the minor groove of the duplex RNA alters tertiary interactions and has been shown to contribute to stacking within the helix, stabilizing it (132, 133, 134). The reaction is catalyzed by pseudouridine synthases (PUSs) in all domains of life, which modify tRNA and rRNA in bacteria and tRNA and snRNA in eukaryotes (135, 136). Eukaryotes and archaea contain the additional enzyme of Dyskerin, which uses snoRNAs to guide modifications in rRNA. Recent evidence suggests that PUS proteins, and in particular PUS1, can pseudouridylate mRNA, and their activity is regulated by stress such as heat shock (124, 125) (Table 2).

Some of the initial studies on the impact of pseudouridylation of mRNA on its translation were conducted by Karikó and Weissman (137). These efforts were, however, motivated by the therapeutic application of the modification and its ability to evade cellular innate immunity. Nevertheless, these studies showed that mRNAs having all of their uridines substituted for pseudouridines are efficiently translated by the ribosome to produce fully functional proteins, suggesting that the modification neither alters the speed nor the fidelity of protein synthesis significantly (137). In contrast to these observations, studies by Karijolich and Yu suggested that pseudouridine-modified stop codons lead to elevated readthrough by promoting misreading of the codon by tRNAs (138). It was suggested that the modification promotes unusual base-pairing interactions (139). More recent studies have exploited this property of pseudouridine modification to suppress premature termination codons *via* engineered snoRNA-mediated pseudouridylation (140, 141, 142). Pseudouridine slightly increases the stability of near-cognate interactions between the codon and anticodon (138). However, one study suggested that this leads to significant miscoding (143), while others have suggested that miscoding is negligible (144, 145). Thus, different groups have made distinct conclusions as to whether the pseudouridine modification alters the fidelity of tRNA selection by the ribosome. Regardless, it is unclear how studies that promote the idea that a mere single pseudouridine can reprogram decoding by the ribosome reconcile the observations that fully pseudouridylated reporter mRNAs, which contain hundreds of pseudouridines, produce fully functional proteins in extracts and in cells. Furthermore, no readthrough events could be detected in these studies (137, 144, 145). The argument that sequence-context plays a major role during recoding does not hold water, since multiple reporters with pseudouridine occupying thousands of contexts have been used so far in these therapeutic-based studies.

## Chemical damage to RNA and its consequences

Similar to what occurs in DNA, nucleobases of RNA are also vulnerable to nonenzymatic chemical modifications due to their reactivity with various cellular metabolites as well as exogenous agents (6) (Fig. 2*D*). In fact, the polymer exhibits a much more pronounced susceptibility to chemical damage relative to DNA (3, 7). This stems from several intrinsic features of RNA, including its abundance, overall structure, and its interactions with proteins or lack thereof. In a typical cell, RNA and its NTP precursors can be more than an order of magnitude more abundant than DNA and its dNTP precursors, respectively (146). Furthermore, unlike the base-paired nature of the DNA helix, RNA is on average 30 to 40% single stranded, exposing the Watson–Crick faces of the nucleobases to reactive chemical species (3). In eukaryotes, DNA is further shielded by its association with histone proteins and its nuclear localization, whereas RNA is more exposed and is directly accessible to damaging agents such as mitochondrial metabolites (6, 147, 148). It is also worth noting that the susceptibility to damage varies across RNA species, in which protein association and secondary structure formation playing key roles in determining the extent an RNA species experiences chemical damage during its lifetime (3, 149). To this end, several studies have shown that mRNA appears to be the target of damage (148, 150, 151, 152, 153, 154). In the following sections, we describe the most prevalent chemical modifications observed in mRNA.

### Oxidation

**Figure 4:**
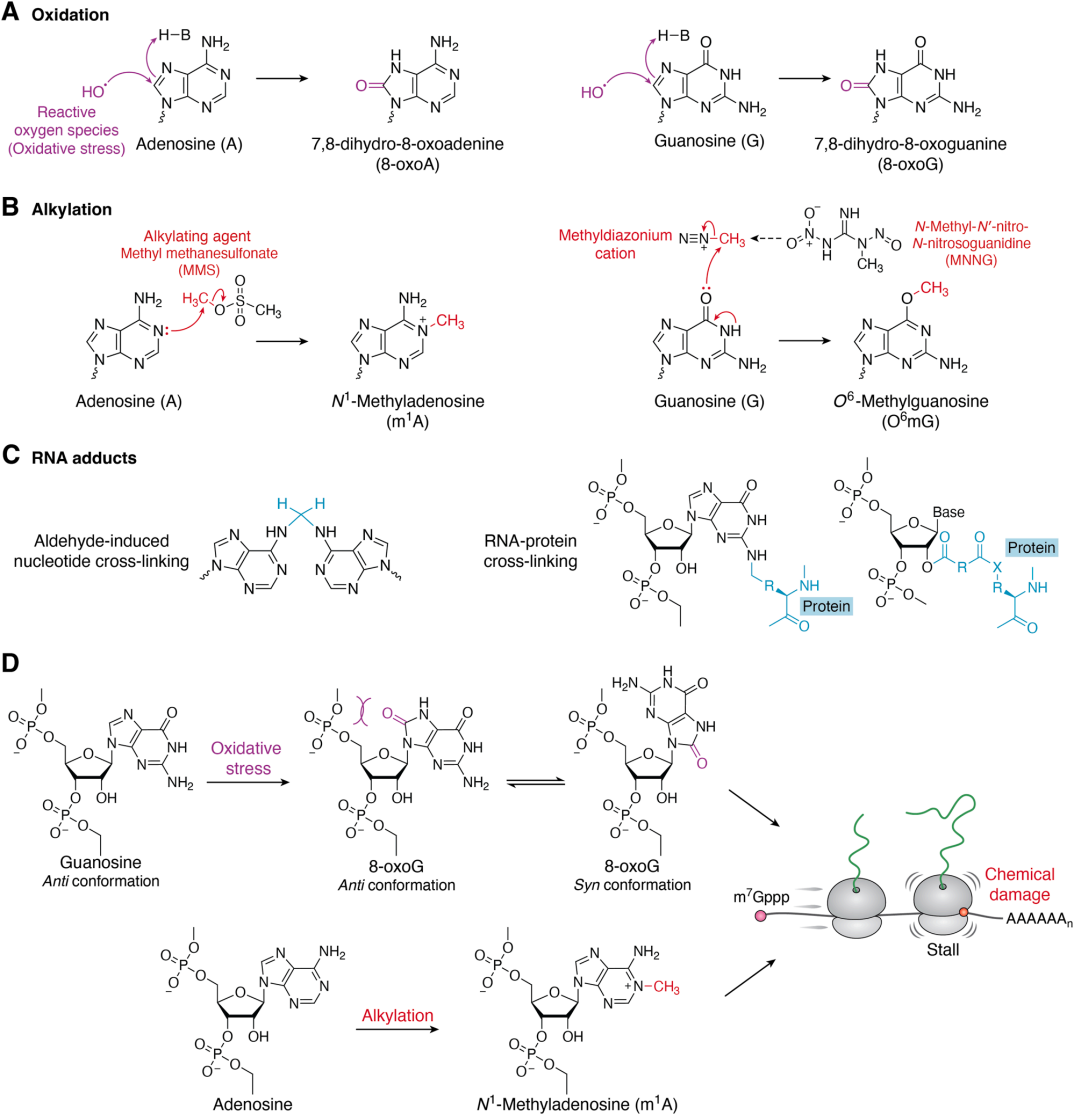
**Chemical damage of nucleobases by ROS and alkylation and crosslinking agents.***A*, oxidation of adenosine and guanosine to 8-oxoadenoinse (8-oxoA) and 8-oxoguanosine (8-oxoG) by hydroxy radicals. *B*, alkylating agents MMS and MMNG react with nucleobases to form N-alkyl adduct N1-methyladenosine (m1A) and O-alkyl adduct O6-methylguanosine, respectively. *C*, RNA adducts formed by aldehyde crosslinking agents and covalent crosslinking of RNA to proteins. *D*, 8-oxoG and m1A inhibit tRNA selection and stall ribosomes. ROS, reactive oxygen species.

Oxidative damage results from reactions between RNA and ROS, which can be byproducts of cellular metabolism as well as the environment. The most significant source of endogenously generated ROS is the mitochondria (155). During cellular respiration, electrons can prematurely leak from the electron transport chain, which can react with oxygen to form the superoxide anion (O_2_^•-^), which is often termed as the primary ROS, subsequently producing much more potent secondary ROS such as hydroxyl radicals, perhydroxyl radicals, and hydrogen peroxide (156, 157). Furthermore, O_2_^•-^ can react with nitric oxide to form the peroxynitrite (158). Regardless of their source, these highly reactive species are potent in attacking the nucleobases (157). Common modifications include 8-oxo-7,8-dihydroguanosine (8-oxoG), 8-oxo-7,8-dihydroadenosine (8-oxoA), diamino-4-hydroxy-5-formamidopyrimidine, 4,6-diamino-5-formamidoadenine, 5-hydroxyuridine, 5-hydroxycytidine, and cytosine glycol (159, 160, 161) (Fig. 4*A*). Among these, 8-oxoG stands out as it is highly abundant because guanine has the lowest reduction potential among the four bases (162). Furthermore, the modification has long been known to be associated with neurodegenerative diseases. Indeed, in early studies on RNA damage, it was shown that 8-oxoG accumulates to very high levels in the mRNA pool of the neurons of Alzheimer’s patients (148, 163, 164). In some of these studies, it was estimated that 50% of cellular mRNAs contain at least one damage (165). However, it remains unclear to what extent RNA oxidation contributes to the pathogenicity of the disease or whether it is a by-product of the disease (14, 150).

Given that the modification does not directly alter the Watson–Crick face of guanine, so how does it affect the base-pairing properties and hence the function of the nucleobase? The introduction of a carbonyl group at the C8 position imposes a steric clash with the phosphate backbone at C5, destabilizing the canonical *anti* conformation (166) (Fig. 4*D*). For decades, we have known that in DNA, the modification results in the nucleotide preferring the *syn* conformation, which alters hydrogen bonding and allows 8-oxoG to form a Hoogsteen base pair with adenosine during DNA replication (166). Whether the modification can similarly alter mRNA–tRNA interactions during translation on the ribosome, and hence produce defective protein products, was initially unclear. Early studies suggested that oxidation of the firefly luciferase mRNA compromised the fidelity of protein synthesis without inhibiting mRNA association with ribosomes (152). However, these studies showed that the yield of protein synthesis is significantly lower in the presence of oxidized mRNA relative to undamaged one (148, 151, 152). It should be noted that these studies utilized bulk oxidized mRNA and as a result the impact of a specific adduct on tRNA selection by the ribosome could not be readily discerned. In one of the first studies, our group utilized a fully reconstituted *in vitro* translation system in combination with synthetic mRNAs containing 8-oxoG at defined sites to reveal that the modification reduces the rate of peptide-bond formation by more than three orders of magnitude (167). Later studies suggested that 8-oxoG nucleobases predominantly adopt the *syn* conformation within the ribosomal A site but is unable to base pair with adenosine to promote miscoding. Indeed, 8-oxoG adduct was found to disrupt the initial phase of tRNA selection, which depends on codon recognition by the tRNA (168). These discoveries were unexpected as they suggested that the ribosome does not follow the same rules as DNA polymerases and that the decoding center is more restrictive to the types of base-pairing interactions it allows between the mRNA and tRNA.

### Alkylation

In addition to oxidative lesions, nucleobases are highly susceptible to modifications by alkylating agents. The endogenous alkylating agent S-adenosylmethionine (SAM) is particularly noteworthy due to its relatively high intracellular concentration (in the millimolar range), resulting in its nonenzymatic reactivity with nucleic acids and other biomolecules (169). Other endogenous alkylating agents include decomposition products of nitrosated amines and bile acids, which form highly reactive lactones that damage nucleic acids (170). Environmental alkylating agents include halogenated hydrocarbons and byproducts of tobacco smoke (171). Arguably, however, some of the most significant exogenous sources of alkylative damage are chemotherapeutic agents, which include cyclophosphamide, streptozotocin, and Temodar (172, 173, 174). These agents modify both DNA and RNA, with recent studies suggesting that modification of the RNA by these drugs contributes to their efficacy (175, 176, 177).

Among the alkylation adducts, O-alkyl modifications are special, because of their ability to be both mutagenic and genotoxic (177). For instance, during DNA replication, O6-methylguanosine readily mispairs with thymidine, forming a geometry that is virtually indistinguishable from a Watson–Crick base pair (178). In contrast, N-alkylation of the Watson–Crick face or the minor groove of the nucleobase typically prevents proper base pairing and recognition by polymerases and hence are only genotoxic (179). In mRNA, O6-methylation was found to promote miscoding by base pairing with uridine in the anticodon but only when found at the first and third position of the codon. It should be noted that G•U base pairs are tolerated at the third position irrespective of O-methylation. In contrast to the first and third position, O6-methylation of guanosine at the second position inhibits tRNA selection altogether (83) (Fig. 4*B*). These observations are consistent with the mechanism by which the second position of the codon is decoded, which requires multiple intricate interactions with the decoding center nucleotides (180, 181). It also suggests that the ribosome can be more “choosy” than replicative polymerases during codon–anticodon recognition. In contrast to O-methylation, N-methylation appears to largely impact tRNA selection as expected. For example, N1-methylation of adenosine (Fig. 4*B*), which blocks base pairing, severely inhibits tRNA selection irrespective of its position within the codon. Interestingly, N1-methylation of guanosine alters decoding similarly but only when the modification is introduced at the first and second position of the codon (182, 183). The distinction between the effects of m1A and m1G on decoding can be attributed to the fact that the former modification is accompanied by the introduction of a positive charge to the base.

### UV irradiation and protein crosslinking

UV damage of nucleic acids is a well-documented biological phenomenon. Early insights into UV-mediated RNA damage came from *in vitro* experiments revealing that RNA treated with UV contains modified nucleotides and dimers (184). It is now generally accepted that UV induces transcriptome-wide RNA damage through photochemical reactions that generate pyrimidine dimers, covalent RNA–RNA, RNA–protein crosslinks, as well as oxidative modifications and other photoproducts (2). The effects of most of these adducts on ribosome function have not been extensively explored but given the dramatic impact lesions such as pyrimidine dimers have on duplex structure (185), it is safe to say that photoproducts are likely to stall translating ribosomes. Indeed, UV irradiation of mammalian cells leads to the accumulation of RNase-resistant disome structures, indicative of ribosome stalling (186). In contrast to our understanding of the cellular response to UV-induced DNA damage (187, 188), the cellular response to UV-induced RNA damage has received much less attention. Nevertheless, as we shall see later, exciting recent data have begun to reveal that UV-induced RNA damage through its effect on translation activates pathways that converge on signals that overlap with those induced in response to DNA damage.

In addition to UV-induced crosslinking, RNA is also vulnerable to other crosslinking agents. This includes formaldehyde, which is a byproduct of some biological reactions such as oxidative demethylation (189) (Fig. 4*C*). Spontaneous and alkylation-induced depurination can also result in crosslink formation between and within RNAs as well as RNA with proteins (190). A major challenge in studying the consequences of RNA crosslinking lesions is that DNA is also frequently affected by these crosslinking agents. Recent work from the Stingele lab overcame this challenge by utilizing the nucleobase analog 4-thiouracil, which is only incorporated into RNA, and has been extensively used to study protein–RNA interactions in photoactivatable ribonucleoside enhancing crosslinking (191). As expected, and given the bulky nature of these adducts, RNA–protein crosslinks were found to stall translating ribosomes but more importantly activate a unique quality control process, which will be discussed later. At the same time of this study, the Beli *et al*. showed formaldehyde-induced RNA–protein crosslinks (Fig. 4*C*) similarly stall the ribosome and activate the same pathway to resolve the impediment and rescue the ribosome (191).

In summary, irrespective of the idiosyncrasies associated with the type of modifications and their position within codon, it is now abundantly evident that damage to mRNA has the potential to profoundly affect ribosome function (Fig. 4*D*). This, in turn, suggests that organisms are likely to have evolved mechanisms to quickly rid their cells of damaged mRNA.

## Quality control of the damaged/aberrant mRNA

Even prior to the work on the effect of mRNA damage on ribosome function, it was already known that aberrant mRNAs are subject to quality control processes that quickly degrade them (13). Notably—and similar to transcription-coupled nucleotide excision repair, which utilizes the stalled RNAP II to recognize lesions that impede its movement along the DNA template (192, 193, 194)—most of these pathways utilize ribosomes to detect the defect in the mRNA (90, 195, 196). The most relevant pathway to our discussion here is no-go decay (NGD), which as the name suggests is activated in response to mRNAs that cause the ribosome not to go. NGD was initially studied in the context of mRNA reporters that harbor sequences and/or structural elements that impede ribosome movement, inhibitory codons, stable stem-loops, and poly A tracts (13, 197). More recent studies have clearly shown that the pathway is activated in response to chemical damage to the mRNA (8). In its absence, nucleotide adducts accumulate on the polymer, and cells become sensitive to damaging agents. In addition to NGD, which is agnostic as to the type of damage that causes ribosome stalling, lesion-specific quality control mechanisms may also exist. In these translation-independent mechanisms, RNA-binding proteins with affinities to a specific modification may target the damaged RNA for degradation.

### Stalled ribosomes are ubiquitinated in response to collisions

**Figure 5:**
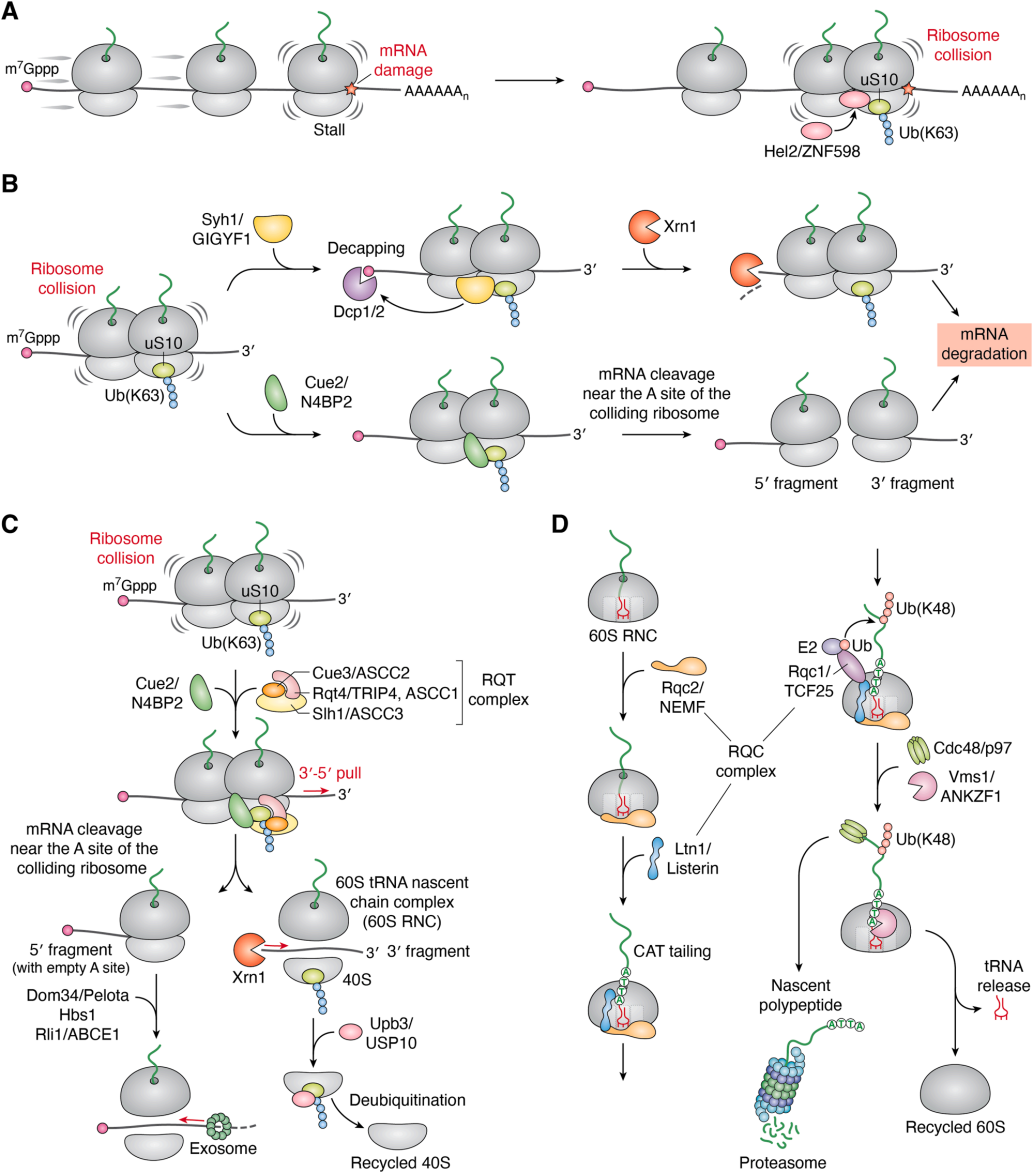
**Ribosome-based quality control of damaged/aberrant mRNA.***A*, mRNA damage stalls ribosomes, leading to their collisions. Collided ribosomes are recognized by the E3 ligase Hel2/ZNF598, which ubiquitinates ribosomal proteins. Shown is its uS10 target. *B*, aberrant mRNAs are degraded through two possible pathways. In the first pathway, Cue2/N4BP2 recognizes ubiquitinated stalled ribosome and uses its SMR domain to cleave the mRNA near the A site of the colliding ribosome. In the second pathway, colliding ribosomes recruit Syh1/GIGYF1, which promotes decapping and subsequent Xrn1-dependent degradation of aberrant mRNAs. *C*, the ubiquitinated stalled ribosomes are recognized by RQT complex composed of Slh1/ASCC3, Cue3/ASCC2, and Rqt4/TRIP4, ASCC1. Cue3 possesses ubiquitin-binding domains that recognizes K63-linked ubiquitin chains, while Rqt4 makes contacts with uS10. In concert with Cue3 and Rqt4, Slh1 binds to mRNA channel and uses its RNA helicase activity to pull on the mRNA to physically disassemble ribosomes triggering NGD and RQC. The 40S containing uS10-Ub is deubiquitinated by Ubp3/USP10 and recycled, while the 60S containing tRNA nascent chain complex (60S RNC) is further subjected to RQC. In a second branch of NGD, Cue2/N4BP2 is recruited to the colliding ribosome and cleaves the mRNA in the A site. The resulting ribosomes with empty A sites are rescued by Dom34/Pelota, Hbs1, and Rli1/ABCE1. The resulting 5′ fragment is degraded by the exosome while the 3′ fragment is degraded by XRN1. *D,* degradation of 60S-RNC by RQC. Recognition of the incomplete peptide attached to the peptidyl-tRNA on the 60S subunit is catalyzed by the RQC complex consisting of Rqc2/NEMF, Ltn1/Listerin and Rqc1/TCF25. In particular, Rqc2 is recruited to 60S-RNC and stabilizes the binding of Ltn1 and adds CAT-tailing to the nascent peptide to expose the lysine residues buried in the ribosomal exit tunnel. At this stage, Rqc1 specifically interacts with the RING domain of Ltn1 and adds K48 Ub chain to nascent peptide. Vms1 then cleaves the acceptor stem of the tRNA, releasing the ubiquitinated peptide. Cdc48 and its cofactors consequently extract the nascent polypeptide and deliver it to the proteasome for degradation. NGD, no-go decay; RQC, ribosome quality control; RQT,RQC-trigger.

Notably, the eukaryotic response to ribosome stalling was initially studied in the context of its consequences to the defective mRNA and the resulting incomplete nascent peptide (13). How stalled ribosomes are identified was not clarified until the discovery that the quality control of the aberrant mRNA and encoded protein is dependent on the E3 ligase Hel2 in yeast (ZNF598 in mammals) (91, 195, 198, 199, 200, 201, 202). Indeed, deletion of the factor was found to result in readthrough of stall sequences and stabilization of stalling mRNA reporters (201, 203, 204). Later studies established that the substrates for the ligase are ribosomal proteins; Hel2/ZNF598 adds K63-linked ubiquitin chains to a number of ribosomal proteins, including uS10, uS3, and eS7 (15, 90, 205, 206). Among these ubiquitination events, uS10 appears to be the most critical one for ribosome quality control (RQC) (205). It acts as a hub for recruiting downstream factors that are required for robust degradation of the defective mRNA and peptide, as well as rescue of the stalled ribosomes (Fig. 5*A*).

Given its sub stoichiometric ratio to ribosomes, how Hel2 identifies a stalling ribosome from a translating one is a challenge. As importantly, transient stalling events are common and play important roles in gene expression, including cotranslational folding and protein targeting (207, 208, 209, 210). Therefore, it is paramount that Hel2 only recognizes ribosomes stalled on problematic mRNAs. A key insight into Hel2’s substrate ribosome specificity came out of our group’s work, in which we showed robust ubiquitination of ribosomal proteins only occurs under conditions that promote ribosome collisions (15). This occurs when the lagging ribosomes run into the leading stalled ribosome. Structural studies from the Hegde, Inada, and Beckman *et al*. showed such a collision results in a unique ribosome–ribosome interface that is likely to be recognized by the E3 ligase (90, 91). The ability of Hel2 to recognize and ubiquitinate collided ribosomes is key for quality control in the presence of RNA damage. The E3 ligase robustly adds ubiquitin chains to ribosomal protein in the presence of oxidizing and alkylating stresses (8, 94, 211). In its absence, not only do nucleotide adducts accumulate in the presence of damaging agents but also protein aggregates accumulate (8). Altogether, over the past few years, we have come to appreciate that Hel2 is responsible for initiating signals responsible for clearing damaged mRNAs (Fig. 5*A*), which in turn maintains proteostasis by preventing their ability to encode for truncated protein products.

### NGD is activated downstream of ribosome stalling

About 2 decades ago, and much earlier to the discovery that stalled ribosomes are ubiquitinated by Hel2, Doma and Parker showed that mRNA reporters that stall ribosomes in yeast are destabilized in a translation-dependent manner (13). It was thought that RNA degradation primarily proceeds through an endonucleolytic cleavage because 5′ and 3′ degradation intermediates accumulate in the absence of 3′-5′ degradation by the exosome and 5′-3′ degradation by Xrn1, respectively. Sequencing of these intermediates mapped the cleavage reaction occurring upstream of the stall sequence. While cleavage of stalling mRNAs appears to robustly take place in yeast, such a reaction is yet to be observed in mammals (212). Recent data suggest RNA degradation is primarily carried out by Xrn1, which appears to be recruited to the defective mRNA by Syh1 (GIGYF1 in mammals) (92, 213, 214) (Fig. 5*B*). In the absence of Xrn1, destabilization of the defective mRNA is accomplished through the endonucleolytic reaction described above, which is catalyzed by Cue2 in yeast (N4BP2 in mammals) (92, 215) (Table 3). The protein is directly recruited to the ubiquitinated stalled ribosome using its N-terminal ubiquitin-binding domains (Cue and UBA domains). Once there, the protein uses its C-terminal SMR hydrolase domain, which is also homologous to the C-terminal domain of the bacterial initiation factor 3, to cleave the mRNA near the A site of the colliding ribosome (92, 215) (Fig. 5*B*). This location of the cleavage is consistent with earlier mapping data, showing it to take place upstream of the primary stalled ribosome (92). Although the role of Cue2 in degrading damaged mRNAs has not been explored, Xrn1 appears to play a major role in destabilizing them; in its absence, adducts such as 8-oxoG and m1A accumulate in polyA-enriched RNA (8).

**Table 3:** Proteins involved in ribosome-based quality control of damaged/aberrant mRNA

Category	Protein/complex	Role/function
No-go decay (NGD)/mRNA degradation	Cue2 (N4BP2 in mammals)	Endonuclease recruited to ubiquitinated collided ribosomes *via* N-terminal Cue/UBA ubiquitin-binding domains; cleaves mRNA near the A-site of the colliding ribosome using its SMR hydrolase domain
	Syh1 (GIGYF1 in mammals)	Recruited to collided ribosomes; promotes decapping and recruits XRN1 for 5′-to-3′ degradation of defective mRNAs
	XRN1	5′ → 3′ exonuclease; principal cytoplasmic enzyme for degrading decapped or endonucleolytically cleaved mRNA; major effector of NGD
	Cytoplasmic exosome	3′ → 5′ exonucleolytic complex; degrades the 5′ mRNA fragment generated by Cue2 cleavage
RQT/ribosome rescue	RQT complex (yeast)/ASC-1 complex (mammals)	Dissociates primary stalled ribosomes from damaged mRNA; yeast: Slh1/ASCC3 (Ski2-like RNA helicase) + Cue3/ASCC2 (ubiquitin-binding) + Rqt4/TRIP4 (zinc finger); mammals add ASCC1; also involved in DNA alkylation repair
	Dom34/Pelota + Hbs1 + Rli1/ABCE1	Rescue trailing ribosomes that run to the end of the truncated mRNA after Cue2 cleavage; structurally homologous to release factors eRF1/eRF3 but lack stop-codon recognition; Hbs1 N-terminal domain probes the mRNA entry tunnel for vacant ribosomes
	Ubp3 (USP10 in mammals)	Deubiquitinase; removes K63-linked ubiquitin chains on the recycled 40S subunit after RQT-mediated splitting
RQC complex (nascent peptide/60S recycling)	RQC complex: Ltn1/Listerin + Rqc2/NEMF + Rqc1/TCF25	Recognizes peptidyl-tRNA-bound 60S subunits post-splitting and targets the incomplete nascent peptide for proteasomal degradation
	Ltn1 (Listerin in mammals)	RING-domain E3 ligase within the RQC complex; ubiquitinates nascent peptide on stalled 60S with K48-linked chains
	Rqc2 (NEMF in mammals)	RQC cofactor; stabilizes Ltn1 on the 60S; performs CAT-tailing (non-templated alanyl/threonyl addition) to expose buried lysine residues for ubiquitination
	Rqc1 (TCF25 in mammals)	RQC subunit; interacts with Ltn1's RING domain; imposes K48 linkage specificity on the ubiquitination reaction
	Vms1 (ANKZF1 in mammals)	Endonuclease; cleaves the CCA end of peptidyl-tRNA on the stalled 60S, releasing the ubiquitinated nascent peptide
	ELAC1	tRNA repair enzyme; removes the 2′,3′-cyclic phosphate left by Vms1 on the cleaved tRNA
	ANGEL2	CCR4-family tRNA repair enzyme; also removes 2′,3′-cyclic phosphate from Vms1-cleaved tRNA
	TRNT1	CCA-adding enzyme; restores the 3′-CCA end to the repaired tRNA, completing tRNA recycling
	Cdc48 (VCP/p97 in mammals)	AAA ATPase; extracts the ubiquitinated nascent peptide from the 60S subunit and delivers it to the proteasome for degradation
Translation-independent quality control	YBX1/YB-1	Y-box RNA-binding protein; role in stress granule dynamics; has affinity for 8-oxoG-containing RNA, though its role in degrading oxidized RNA remains uncertain
	PNPase (polynucleotide phosphorylase)	3' → 5' exonuclease (bacterial/mitochondrial); displays specificity for 8-oxoG-containing RNA; knockdown in HeLa cells causes accumulation of oxidized RNA and sensitizes to oxidative stress
	AUF1 (hnRNP D)	RNA-binding protein; preferentially binds 8-oxoG-containing mRNA; recruits CCR4-NOT deadenylation complex to trigger rapid degradation of oxidized transcripts
	CCR4-NOT	Deadenylase complex; interacts with AUF1; promotes deadenylation and degradation of oxidized mRNA
	PCBP1	RNA-binding protein; recognizes RNA containing two closely spaced 8-oxoG residues; binding linked to induction of apoptosis

### RQC trigger dissociates the two subunits following ribosome stalling

Although defective mRNAs and their encoded peptides pose risks to maintaining cellular proteostasis, their ability to retain multiple ribosomes and keep them away from the translating pool is arguably much more deleterious to cellular homeostasis. It is not a surprise then that RQC and NGD are intimately coupled to downstream ribosome rescue through a dissociation reaction of the two subunits off the defective mRNA (197, 205, 216, 217). Early genetic studies in yeast identified the key players in this process (13, 201), while recent biochemical and structural studies have provided important insights into how these factors promote ribosome dissociation and recycling of the subunits (218, 219, 220). At least two distinct recycling pathways appear to take place following stalling. On trailing ribosomes that run to the end of the mRNA following cleavage by Cue2, Dom34 (Pelota in mammals) and Hbs1 work in concert with the canonical recycling factor Rli1 (ABCE1 in mammals) to facilitate ribosome dissociation (217, 218, 221, 222) (Fig. 5*C*). Dom34 and Hbs1 are homologous and are structurally related to release factors eRF1 and eRF3, respectively (223). However, Dom34 lacks the key domains that allow eRF1 to recognize stop codons and catalyze peptide release in the active site of the ribosome, consistent with the idea that the factor should display no mRNA sequence preference. The question of how the Dom34/Hbs1/Rli1 complex is able to identify ribosomes that have little to no mRNA downstream of the P site was addressed using structural studies that showed the N-terminal domain of Hbs1 to bind in the mRNA entry tunnel (217, 218, 219, 222) (Table 3). As such, the factor is unable to dissociate normal translating ribosomes with their entry tunnel occupied by mRNA; a feature shared by the tmRNA/SmpB rescue pathway in bacteria (224).

On the other hand, RQC-trigger (RQT) complex containing the ATP-dependent Ski2-like RNA helicase Slh1 (ASCC3 in mammals) dissociates the primary stalled ribosomes (Fig. 5*C*) and unlike Dom34/Hbs1/Rli1 requires an intact mRNA with an accessible 3′ end (225, 226). In yeast, the RQT complex consists of Slh1, the ubiquitin-binding CUE domain containing protein Cue3 (ASCC2 in mammals), and the zinc-finger containing protein Rqt4 (TRIP4 in mammals) (225) (Table 3). Humans contain the additional protein ASCC1, which together with the other factors form the ASC-1 complex (226). Cue3 is thought to use its ubiquitin-binding domains to bind K63-linked ubiquitin chains on the ribosome and recruit the splitting complex to stalled ribosomes (216). In agreement with these ideas, the structure of the complex with ribosomes revealed that Cue3 in concert with Rqt4 bind near uS10 (Fig. 5*C*), which is a major substrate for Hel2-mediated ubiquitination and is required for downstream initiation of RQC (227). Once the RQT complex is on the ribosome, it places its key factor Slh1 near the entry tunnel of the ribosome, which uses its ATP-dependent helicase activity to pull on the mRNA. The pulling motion causes the head of the small subunit of the stalled ribosome to swivel and the trailing ribosome to move forward. This creates a clash between the large subunits of the collided ribosomes, forcing dissociation of the stalled ribosome (216). Emerging evidence suggests that ribosome splitting on damaged mRNA is critical upon exposure to damaging agents. In particular, the inability of ASC-1 complex to rescue ribosome stuck on bulky mRNA adducts results in cell cycle arrest through activation of the ribotoxic stress response (228).

### RQC recycles the large subunit and targets the nascent peptide for proteasomal degradation

Splitting of collided ribosomes by the RQT (ASC-1) complex results in a free 40S subunit, which is ready to be reused for translation. On the other hand, peptidyl-tRNA bound 60S subunit must be processed in order for it to be completely recycled (229). In particular, the incomplete peptide is subjected to ubiquitination and proteasomal degradation—whereas following its ejection, the tRNA is also recycled. One of the key players in the process is the E3 ligase Ltn1 (Listerin in mammals) (230, 231, 232), which is responsible for ubiquitinating the nascent peptide on the large subunit. Together with its cofactor Rqc2 (NEMF in humans), it distinguishes peptidyl-tRNA-bound large subunit from ones engaged in translation by making interactions at intersubunit bridges that are only possible in the absence of the small subunit (195, 230, 231, 232). Rqc2 has the additional activity of nontemplated addition of alanyl and threonyl residues to the C terminus (233, 234). This so-called process of CAT tailing has been suggested to position a required lysine residue in the active site of Ltn1 (235, 236). Alternatively, in the absence of lysine residues altogether, CAT-tailing by Rqc2 can function as a degradation tag in the absence of ubiquitination (237). Others have argued that it can also lead to aggregation of the nascent peptide (234, 238). In addition to Ltn1 and Rqc2, the complex contains Rqc1 (TCF25 in humans), which interacts with the RING domain of Ltn1 (Listerin) and imposes K48 specificity on the ubiquitination reaction (239). Nascent peptide extraction is achieved by the AAA ATPase Cdc48 (VCP in mammals) and its cofactors (240, 241) following an endonucleolytic cleavage of the CCA end of the peptidyl tRNA by Vms1 (ANKZF1 in mammals) (242, 243) (Fig. 5*D*) (Table 3). Trimmed tRNA is first healed by ELAC1 and ANGEL2, which remove the 2′,3′ cyclic phosphate group left by Vms1 before CAA can be added by TRNT1, completing the recycling of the tRNA (244, 245, 246). Highlighting the significance of RQC-mediated degradation of incomplete peptides that result from translation of damaged mRNA, deletion of Ltn1 leads to the accumulation of protein aggregates in the presence of oxidizing and alkylating agents. Furthermore, the inability to extract these incomplete peptides from the large subunit by Cdc48 increases the ubiquitination of nascent peptides in response to RNA alkylation and oxidation (8).

### RNF14 and RNF25 E3 ligases are required for resolving ribosomes stalled on mRNA protein crosslinks

UV- and aldehyde-induced mRNA protein crosslinks within the CDS region of the mRNA introduce a roadblock to translation that is in principle similar to that experienced on chemically modified mRNA with one important distinction. On one hand, they cause stalling and eventual collisions, which in turn activate RQC to rescue the stalled ribosomes and target the nascent peptide for degradation (191). On the other hand, the crosslinked protein presents an impediment to the RNA degradation machinery downstream, and as such needs to be removed. Two groups showed that under these conditions, two E3 ligases are recruited to the ribosome in human cells in order to resolve mRNA protein crosslinks (191, 247). RNF25 ubiquitinates ribosomal protein eS31, which is necessary for the ubiquitination of the crosslinked protein by RNF14. The ubiquitinated protein is extracted by VCP/p97 (Cdc48 in yeast) and presented to the proteasome for degradation (248). Any RNA remnants should be readily degraded by Xrn1 or the cytoplasmic exosome. It should be noted that RNF25 and RNF14 are recruited to the ribosomes by GCN1 (191), which has recently emerged as a key sensor of collided ribosomes (249). For instance, it recruits RNF14 to ubiquitinate elongation and release factors stuck in the A site of the ribosome (250, 251). More importantly, and as we will discuss later, it is required for GCN2 activation and downstream induction of the integrated stress response (ISR) in response to chemical damage to the mRNA.

## Translation-independent quality control mechanism

Many physical and chemical agents that damage RNA also activate stress pathways that inhibit translation initiation and promote eventual polysome dissociation (3). In the absence of scanning ribosomes, alternative translation-independent mechanisms might be required to recognize and degrade damaged RNA. Notably, inhibition of translation allows for extensive RNA–RNA and RNA–protein intermolecular interactions, which can lead to the assembly of liquid-like mRNA-rich condensates in the cytoplasm known as stress granules (252). The RNA-binding protein YB-1 or YBX1—a member of the Y-box-binding protein family in mammals—appears to play an important role in stress granule dynamics (253, 254). In addition, YB-1 has been suggested to have a higher affinity for 8-oxoG containing RNA, but a possible role for the factor in degrading oxidized RNA is yet to be shown (255). Similarly, *Escherichia coli* polynucleotide phosphorylase, which primarily functions in exonucleolytic degradation of RNA in the 3′ → 5′ direction, appears to display a specificity for 8-oxoG-containing RNA and has been suggested to play a possible role in removing oxidized RNA (256). Indeed, knockdown of polynucleotide phosphorylase in HeLa cells was found to result in the accumulation of oxidized RNA and sensitize cells to oxidation stress (257). By contrast, its overexpression was protective against RNA oxidation and cellular death upon the addition of hydrogen peroxide. AU-rich element RBP 1 (also known as hnRNP D) has been suggested to preferentially bind to 8-oxoG-containing mRNA (258). Since the protein is known to interact with the CCR4-NOT deadenylation complex (259), it has been suggested that AUF1 binding to oxidized RNA is likely to trigger their rapid degradation (258). Finally, a more recent study also identified poly(rC)-binding protein 1 (PCBP1) as an 8-oxoG reader protein. However, unlike AUF1, which recognizes a single 8-oxoG, the binding of PCBP1 to RNA requires two 8-oxoGs located in close proximity, which could only occur under highly destructive oxidative stress. As such, it has been suggested that the binding of PCBP1 to oxidized RNA is somehow responsible for induction of apoptosis under oxidative stress (260). It should be noted, however, that all of these RBPs have established functions in RNA metabolism beyond their supposed role in recognizing oxidized RNA. In particular, they recognize specific sequences/structure within RNA, and it is unclear how this function overlaps with their potential ability to recognize 8-oxoG.

What about other adducts? Are there RNA-binding proteins that display a specificity toward them? For m1A, several groups suggested that the YTH class of RBP (YTHDF1-3 and YTHDC1) display specificity toward m1A-containing RNAs (261, 262, 263, 264). In turn, binding of these proteins can accelerate the degradation of the target modified RNA. These findings were not without controversy, as these proteins are known to bind m6A-containing RNA with much better affinities. Others have argued, given the impact of m1A on duplex RNA structures, these studies do not report on RNA modification *per se* but instead on the unwinding of the RNA structure. Indeed, structural studies have shown that the YTH proteins have a binding pocket that is tailored for m6A (265, 266), which is unlikely to accommodate m1A with its positive charge. Additionally, given that m1A can undergo conversion to m6A (267), albeit under elevated temperature and pH conditions, it has been suggested that YTH proteins binding to m1A containing RNA may reflect its conversion to m6A under experimental conditions. Regardless of whether there are *bona fide* "readers" for damaged mRNAs, it is less likely that organisms have evolved a specific protein factor for recognizing each adduct that can accumulate in RNA. Instead, it is more likely that cellular pathways for degrading damaged mRNA act in a translation-dependent manner while being independent of the specific type of lesion.

## mRNA damage in signaling

Although quality control processes are widespread in biology and play important roles in removing defective molecules, they are often not sufficient for maintaining cellular homeostasis in response to chemical and physical insults. This is particularly true for how cells respond to DNA damage, for example. On one hand, they activate the appropriate DNA repair pathway; but on the other hand, if the damage is relatively severe, they activate signaling pathways through protein kinases to arrest the cell cycle and, if necessary, induce cell death (11). Interestingly, the key signal for these kinases is DNA damage itself, such as accumulation of ssDNA or dsDNA breaks (11). In exciting new reports, several groups have found that organisms use damaged mRNA to activate stress signaling pathways through their impact on ribosome function (93, 94, 186). These processes allow cells to reprogram gene expression and adapt to stress conditions. More recent studies have even established that damaged RNA regulates cellular fates and can trigger apoptosis in response to severe stress conditions (93). Emerging from these studies is that cells carefully monitor the frequency of ribosome collisions to activate the appropriate response (268).

### The ISR is activated by collided ribosomes in response to mRNA damage

We have known for almost 50 years that eukaryotes globally regulate protein synthesis in response to stress through the activation of the ISR. Initially discovered in mammalian cell extracts, eIF2α was found to be phosphorylated by protein kinases in response to heme unavailability, the presence of double-stranded RNA, or oxidative stress (269, 270, 271). Later studies in yeast showed that the initiation factor can also be phosphorylated by Gcn2 in the absence of amino acids (272). Phosphorylation of eIF2α at serine 51 has profound effects on PIC formation as it renders the protein a competitive inhibitor for its guanine exchange factor eIF2B, effectively limiting the concentration of the initiator tRNA ternary complex (45, 273, 274, 275, 276). Consequently, overall translation output is inhibited; however, prosurvival mRNAs (such as Gcn4 and ATF4 in yeast and humans, respectively) that undergo atypical initiation mechanisms are preferentially translated under these conditions (268, 272, 277). We now know that mammals have four eIF2α kinases: PKR, HRI, GCN2, and PERK, which are activated in response to ds-RNA, cytosolic protein misfolding, ribosome collisions, and ER stress, respectively (278, 279, 280) (Table 4). Among these kinases, GCN2 is the most widespread and found nearly in all eukaryotes. Its mode of activation has been heavily studied in yeast, in which it is the only known eIF2α kinase (275, 281). Gcn2 was initially studied in the context of amino acid deprivation and in particular histidine starvation. Notably, analysis of the domain architecture of the protein revealed that it has a histidyl tRNA synthetase–like domain similar to the bacterial RelA protein, which activates the stringent response, inhibiting transcription, and translation through the production of the alarmones (p)ppGpp (282, 283, 284, 285). RelA uses its Thr-RS-like domain to sense deacylated tRNAs in the A site of the ribosome, which accumulate under amino acid starvation conditions (286). Analogous to the ISR, the stringent response also reprograms gene expression, increasing amino acid biosynthesis and stress response and inhibits proliferation (287). These similarities between the two processes were used to come up with an initial model by which Gcn2 is likewise activated by deacylated tRNAs on the ribosome (288, 289, 290, 291). Supporting this model were the observations that its cofactors Gcn1 and Gcn20 bind ribosomes (285, 292, 293, 294) and that mutations in its histidyl tRNA synthetase–like domain abrogate its activation (295). Therefore, it was proposed that Gcn1 and Gcn20 deliver a deacylated tRNA to Gcn2 on the ribosome, leading to the dimerization of the kinase and activation through autophosphorylation.

**Table 4:** Factors involved in mRNA damage response and stress signaling

Category	Protein/complex	Role/function
Ribosome collision sensing	ZAKα (MAP3K20 long isoform)	MAPKKK; senses collided ribosomes *via* sterile alpha motif domain dimerization; anchors to the disome through RACK1, ribosomal protein eS27, and 18S rRNA; activates p38 and JNK
	Hel2 (ZNF598 in mammals)	E3 ubiquitin ligase; recognizes the unique interface of collided ribosomes; adds K63-linked ubiquitin chains to ribosomal proteins uS10, uS3, and eS7; initiates both RQC and NGD
	GCN1 (Gcn1)	Large HEAT-repeat collision sensor; recognizes collided and stalled ribosomes; recruits GCN2 for ISR activation and RNF14 for mRNA–protein crosslink resolution
	RACK1	Core ribosomal protein of the small (40S) subunit; required for ZAKα activation during RSR and for ribosome-associated quality control
	Mbf1 (EDF1 in mammals)	Binds collided ribosomes; prevents stalling-induced frameshifting; required for robust GCN2-mediated eIF2α phosphorylation under stress conditions
Integrated stress response (ISR)	eIF2α	Subunit of eIF2 ternary complex; phosphorylated at Ser51 by ISR kinases (PKR, HRI, GCN2 and PERK in mammals) (Gcn2 in yeast)
	Gcn2 (GCN2 in mammals)	eIF2α kinase; activated by collided ribosomes (primary model) *via* GCN1/Mbf1, and by deacylated tRNAs; relocates to collided ribosomes upon stress
	Gcn20	Coactivator of GCN2; binds ribosomes; required for full GCN2 activation along with GCN1
	GCN4/ATF4	Transcription factors (yeast/human) preferentially translated during ISR *via* uORF-mediated mechanism; promote pro-survival gene expression reprogramming
Ribotoxic stress response (RSR)	p38 (MAPK)	Stress-activated protein kinase downstream of ZAKα; mediates cell-cycle arrest in response to ribotoxic stress
	JNK (c-Jun N-terminal kinase)	Stress-activated protein kinase downstream of ZAKα; mediates apoptotic signaling in response to ribotoxic stress

Abbreviations: RACK1, receptor for activated kinase 1.

As appealing this model was, it could not explain later observations showing that Gcn2 can be activated by conditions that are not necessarily accompanied by accumulation of deacylated tRNA. In yeast alkylating and oxidizing agents such as MMS and 4-NQO, respectively, trigger Gcn2-dependent eIF2α phosphorylation without apparent increase in deacylated tRNAs (94). We now know that MMS and 4-NQO damage mRNA inhibit tRNA selection and eventually lead to ribosome collisions (8). This connection between ribosome stalling and Gcn2 activation in fungi is bolstered by observations that mutations that alter tRNA modifications, which slow down translation, also lead to Gcn2 activation without apparent increase in the levels of deacylated tRNAs (296, 297). At the same time, it was revealed that mammalian GCN2 can be activated in a deacylated tRNA–independent manner, in which depletion of a tRNA and inhibition of ribosome rescue in mice neurons caused ribosome stalling and eIF2α phosphorylation by GCN2 (298). Later studies established that GCN2 is more robustly activated by the P stalk of the ribosome relative to deacylated tRNAs (299, 300). It should be noted that this region of the ribosome interacts with ternary complexes and eEF2 during elongation (299, 300). As a result, its availability is likely to signal stalled ribosomes that poorly interact with elongation factors. Indeed, addition of antibiotics that inhibit tRNA selection as well as depletion of release factor also activate Gcn2 (94). These observations were used to make the argument that an empty A site on the ribosome is required for Gcn2 activation, as compounds that stall the ribosome but keep the site occupied do not activate the kinase.

**Figure 6:**
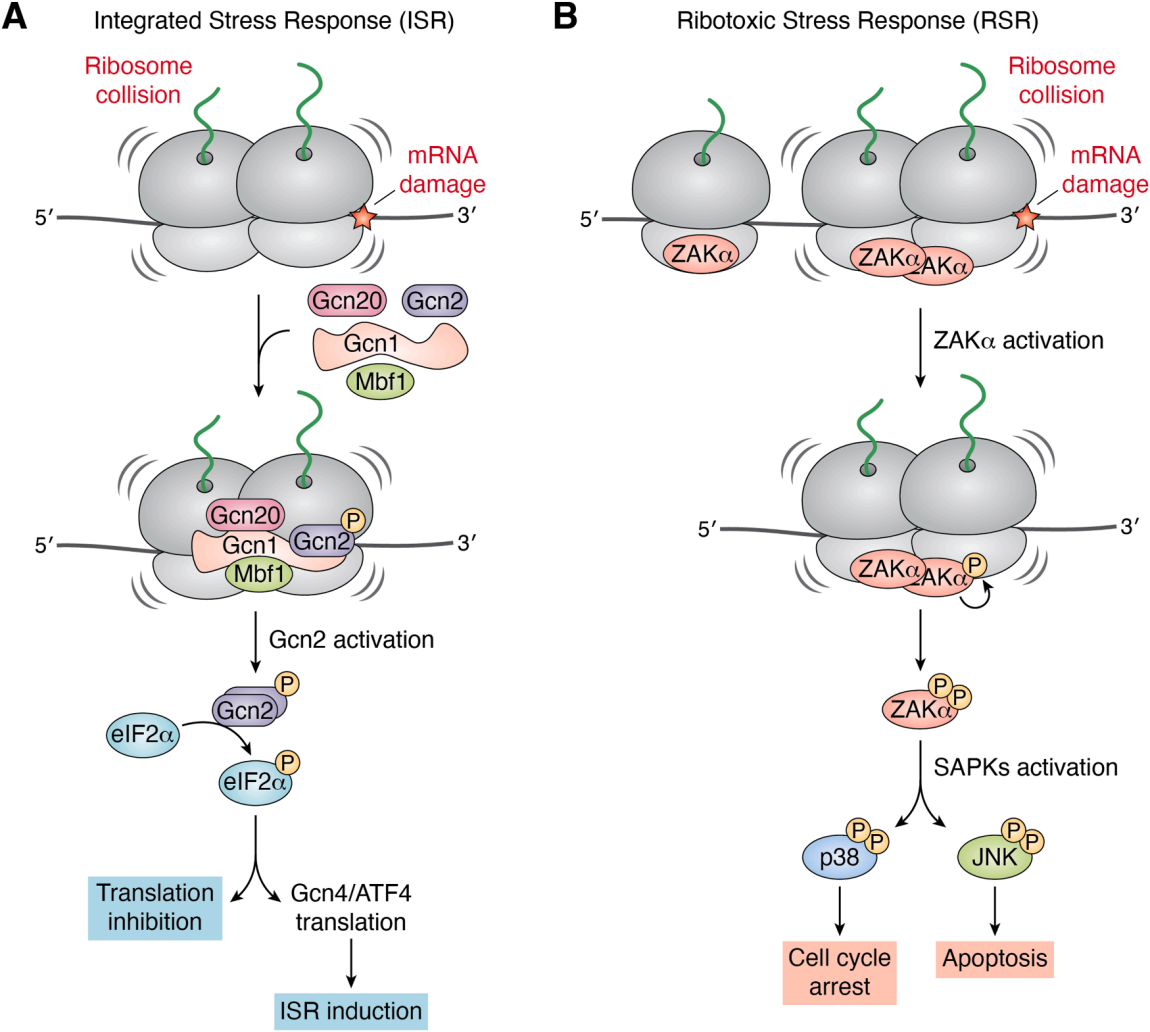
**mRNA damage response in stress signaling.***A*, activation of the integrated stress response (ISR) by collided ribosomes. Gcn2, together with its co-activators Gcn1, Gcn20, and Mbf1 are recruited to collided ribosomes. Activated Gcn2 phosphorylates eIF2α, leading to global translational repression while promoting the selectively translation of stress-adaptive mRNAs such as GCN4/ATF4, triggering the ISR. *B*, the ribotoxic stress response (RSR) is similarly activated by collided ribosomes. MAP3K ZAKα associates with elongating ribosomes and is activated by autophosphorylation in response to ribosome collision. This triggers the RSR by stimulating SAPKs such as p38 and JNK, ultimately promoting cell-cycle arrest or apoptotic signaling. JNK, c-Jun N-terminal kinase; SAPK, stress-activated protein kinase.

The stalling model for Gcn2 activation presented two important questions: (1) How does the factor and its coactivators recognize stalled ribosomes? (2) How does the cell decide between activating the ISR and RQC in response to stalling. Although we are yet to completely answer the first question, studies from multiple groups have provided compelling evidence to suggest that collided ribosomes activate Gcn2. Related to this review, the Green *et al.* and ours, for example, showed that UV, alkylation, and oxidation stresses activate the kinase, and these are accompanied by the accumulation of RNase-resistant disome structures indicative of ribosome collisions (94, 186). Furthermore, optimum eIF2α phosphorylation is observed in the presence of intermediate concentrations of drugs, which stall only a subset of ribosomes, causing widespread collision (94, 186). Consistent with these observations, in a cryo-EM structure, the Wilson *et al*. showed that Gcn2’s coactivator Gcn1 bind collided ribosomes (301). The very large protein uses its extensive HEAT repeats to “snake” itself from the collided ribosome to the stalled one. And although these structures were of a Gcn2-inactive conformation of the collided ribosomes, they revealed how their structure acts as a platform for Gcn1 and potentially activating Gcn2 under the appropriate conditions. Notably, recent cryo-EM studies from the same group revealed that Gcn2 can exist as an inactive dimer bound to the ribosomal 60S subunit, independent of its coactivators, representing a “stand-by” state (302). Upon cellular stress, however, Gcn2 rapidly relocates from the 60S subunit to colliding ribosomes, a process that requires its co-activators, Gcn1 or Gcn20. This observation suggests that association with the 60S subunit may serve as a platform, positioning Gcn2 for quick recruitment to collided ribosomes and enabling rapid activation of the ISR (Table 4). It should be noted that others have argued that there are perhaps two mechanisms for Gcn2 activation, a collision-dependent and collision-independent one, with the latter dependent on Gcn2’s ability to bind to deacylated tRNA (303, 304). However, given that reduction in tRNA aminoacylation would almost always lead to ribosome collisions, the evolution of two independent mechanisms would seem unnecessary. More recent data from our group, in which we showed that robust Gcn2 activation requires the presence of an additional factor Mbf1, provided further support for the collision-dependent model (305). The protein binds collided ribosomes, for which structural studies revealed the mechanism by which it interacts with the colliding and stalled ribosomes and prevents stalling-induced frameshifting. Mutations that inhibit recruitment of Mbf1 to collided ribosomes reduce Gcn2-mediated eIF2α phosphorylation under all tested conditions, including those that induce RNA damaging and amino acid starvation (Fig. 6*A*). A recent study, in which GCN2 activation was reconstituted, provided direct evidence for the ribosome-centric mode of activation. GCN2-mediated phosphorylation of eIF2α under amino acid-starvation conditions only occurred when ribosome collisions were induced, and this activity requires the presence of the collision sensor GCN1 (306). In particular, Zhou *et al*. used an mRNA reporter that harbored proline codons at defined positions. Starving for proline only activated GCN2 when its codons were placed downstream and hence allowing for the accommodation of multiple ribosomes upstream of the stall. Furthermore, the authors found that alkylated mRNA robustly activates GCN2 and its recruitment to collided ribosomes. This result is similar to what we and others have proposed for how alkylation and oxidation stresses activate GCN2 through ribosome collisions *in vivo*. Also, in agreement with our model that the A site of the ribosome needs to be empty for robust GCN2 activation, Zhou *et al.* found that drugs that occlude the A site fail to activate the kinase (306). Interestingly, eIF2α phosphorylation appears to increase under amino acid starvation conditions when deacylated tRNAs cognate to the stalling A-site codon are added.

The answer to the second question of how cells decide between activating RQC and the ISR was motivated by the independent observation from the Guydosh *et al.* and ours showing that deletion of *HEL2* in yeast, and hence abrogation of RQC resulted in increased activation of the ISR (94, 307). Later data from our group showed that while RNA damage leads to the induction of both pathways, inhibition of one results in the overactivation of the other (211). It should be noted that Hel2-mediated ubiquitination of ribosomal proteins appears to be more sensitive to the level of damage relative to Gcn2-mediated phosphorylation of eIF2α, requiring much less concentration of alkylators for robust activity (94, 211). *A priori,* the simplest model that could explain these observations is that Hel2 and Gcn2 and/or its coactivators are in direct competition of each other on collided ribosomes. Inconsistent with this model, the introduction of active-site mutations in Hel2, which do not impact the association between the E3 ligase and ribosomes, increased Gcn2 activation that was observed in a null mutant. Similarly, mutations of Hel2’s target lysine residues in uS10 to arginine, which inhibit RQC, resulted in a similar phenotype of increased induction of the ISR (211). Collectively, our data argued for a more appealing model—in which the downstream events of RQC of ribosome rescue, which depend on Hel2-mediated ubiquitination ribosome, reduce the concentrations of collided ribosomes, which are required for Gcn2 activation. On the other hand, activation of the ISR in response to chemical damage inhibits ribosome loading on damaged mRNAs, similarly reducing the concentration of collided ribosomes and hence Hel2-mediated ubiquitination of ribosomal proteins. Indeed, introducing an alanine at serine 51 of eIF2α, which inhibits its phosphorylation by Gcn2, was found to increase Hel2’s activity in response to chemical damage (94). Notably, and although both pathways are activated in response to damage, they appear to require different conformations of the lead ribosome. Hel2 has no preference for the status of the A site, and Gcn2 and/or its coactivator prefers stalled ribosomes with an available A site. Furthermore, Hel2 appears to have a higher affinity for collided ribosomes to activate RQC, but its concentrations are limiting for the process and as a result is exhausted under high levels of damage, allowing the activation of the ISR when it is absolutely required (94).

### mRNA damage regulates cell fate through the activation of the ribotoxic stress response

The ISR starts as a prosurvival pathway, preserving cellular resources and reprogramming gene expression to restore homeostasis (278). However, activation of GCN2 can also lead to cell-cycle arrest and can even induce apoptosis (308, 309). It should be noted that this appears to occur only when the stress conditions persist. Oftentimes, the stress conditions on ribosome function are so severe to start with, that it does not make sense for cells to wait for the ISR to arrest cell cycle. Under these conditions, mammals (and other metazoans) activate the ribotoxic stress response (RSR). More than 4 decades ago, it was shown that the inhibition of ribosome function stabilizes the transcript of the immediate early gene *c-fos*, which is typically induced in response to various stress conditions (310, 311). In 1995, Zinck *et al.* showed that the ribosome inhibitors anisomycin and cycloheximide—which inhibit the peptidyl transferase center (PTC) and translocation, respectively—robustly activate mitogen-activated protein kinase (MAPK) and stress-activated protein kinase pathways (312). These, in turn, are responsible for the downstream induction of the *c-fos* mRNA. They also noted that anisomycin was a much better inducer of these pathways. Two years later, Iordanove *et al.* coined the term ribotoxic stress response when they showed that many agents that compromise the integrity of the ribosome, particularly the PTC and GTPase-activation center on the large subunit, all activated the SAP/c-Jun N-terminal kinase (JNK) (313). Remarkably, they also revealed that activation of JNK by these agents requires translating ribosomes, which suggested that the upstream kinase (a MAPKKK) must somehow recognize disruptions to translating ribosomes. We now know that the RSR activates p38 and JNK, triggering cell-cycle arrest and apoptosis, respectively (93, 186) (Fig. 6*B*) (Table 4). In addition to antibiotics, agents that can damage nucleic acids activate the RSR; these include chemotherapeutic agents and UV irradiation (313, 314). Highlighting the idea that RSR activation is mediated by RNA damage and not DNA is the observation that MAPK activation by UV irradiation still takes place in enucleated cells (93).

A long-standing question in the field was what senses ribotoxic stress and how it is sensed. Although many protein–kinase candidates were initially thought to trigger the RSR, Wang *et al.* were the first to identify the MAPKKK ZAK as the protein responsible for activating p38 and JNK during the RSR (315). Its chemical inhibition or siRNA-mediated knockdown inhibited the induction of the RSR in response to anisomycin and UV. In 2020, two groups independently showed that the long isoform of the protein, termed ZAKα, senses stalled ribosomes (186, 316). However, while one group argued that solitary stalled ribosomes are sufficient to activate the kinase, the other group provided data showing that the factor requires collided ribosomes for its activation. Indeed, Zakα activation of p38 and JNK was found to occur under intermediate concentrations of anisomycin but not under high concentrations of the drug (186). Furthermore, ZAKα was found to associate with the disome structure brought about by the intermediate concentrations of anisomycin. Consistent with these observations, recent cryoEM structures revealed that ribosome collisions induce dimerization of the protein through its sterile alpha motif domains (317). In particular two ZAKα molecules form a bridge between the collided and stalled ribosomes through interactions with the ribosome-associated receptor for activated kinase 1 and ribosomal protein eS27 and the 18S rRNA. Receptor for activated kinase 1 is an intriguing protein, initially studied in the context of PKC signaling, but it has since been established to be a core ribosomal protein of the small subunit, playing critical roles during RQC as well as reading-frame maintenance in response to stalling. Collectively, these interactions form a finely tuned mechanism ensuring that ZAKα is activated specifically in response to ribosome collisions, avoiding inadvertent activation that could lead to unnecessary stress responses and cellular damage. Overall, these intricate interactions at the molecular level orchestrate the precise dimerization of ZAK's sterile alpha motif domains, a process indispensable for its kinase activation and subsequent signaling cascade in response to ribosome collisions.

The initial study by Wu *et al.*, describing activation of ZAKα by ribosome collisions, also provided important insights into the identity of the damaged RNA species responsible for activation of ZAKα. Transfection of UV-damaged mRNA reporter was sufficient to activate the RSR, suggesting that direct damage to ribosomes is unlikely to be sensed by the kinase (186). More recent data from the same group showed that high doses of UV mediate apoptosis through activation of the RSR and not the DNA damage response (DDR) (93). These observations suggest that at this dose of UV, perturbation to translation presumably due to the accumulation of damaged mRNA precedes DNA damage and as a result signaling through stalled ribosomes dominates.

## Concluding remarks

As outlined throughout this review, recent advances in high-resolution mass-spectrometry and next-generation sequencing techniques have made it increasingly clear that mRNA is subject to a myriad of enzymatic and chemical modifications. By altering the structure of the nucleobases, these modifications expand the chemical diversity of the molecule and have the potential of introducing new layers of regulation. Modifications could impact mRNA stability, localization, translational efficiency, and recoding. Indeed, it is without a doubt an exciting time to be studying how alterations to the nucleobases of the molecules impact their function and interaction with other biomolecules. At the same time, given the exquisite sensitivity of some of these methods, researchers need to be careful in distinguishing between “real” mRNA modifications from biological “noise”, resulting from off-target enzymatic reactions. Furthermore, data generated by high-throughput techniques need to be interpreted with caution. The complexity of m6A modification illustrates this challenge, with earlier studies suggesting that the modification within the CDS enhances translation (87). More recent work suggests that it slows down translation and promotes cotranslational mRNA decay (CDS-m6A decay) (81). Furthermore, interpretations must account for potential false positives, particularly when comparing cell systems that differ in the enzymatic machinery responsible for installing and removing these modifications.

In addition to enzymatic modification of the molecule, mRNA is subject to chemical damage from reactive cellular metabolites as well as exogenous agents. Chemical modifications induced by alkylating agents and oxidizing agents, for example, generate lesions such as m1A and 8-oxoG in mRNA, respectively, which are highly disruptive to the function of the molecule. Indeed, biochemical characterization of the modifications has shown them to inhibit tRNA selection by the ribosome (168). In cells, mRNA damage has been associated with ribosome stalling and eventual collisions (8, 15, 167). Recent evidence has suggested that cellular homeostasis critically depends on the ability of organisms to respond to mRNA damage through its consequences on ribosome function. Collided ribosomes trigger RQC, which leads to the degradation of the mRNA and the encoded peptide and ribosome rescue. Inability to activate these pathways in response to RNA-damaging agents compromises cellular proteostasis. Consistent with these observations, mRNA oxidation has been linked to several neurodegenerative diseases (14, 148, 165). Furthermore, the efficacy of some chemotherapeutics has been proposed to be the result of their ability to modify cellular RNA. Consistent with these arguments, a very recent study suggested that targeting the RQC pathway can sensitize cancer cells to specific chemotherapies (318). It will be interesting to follow up on these studies and see if these interventions can make it to the clinic.

Whereas the activation of quality control pathways in response to mRNA damage is arguably intuitive, the ability of aberrant mRNA to reprogram gene expression and regulate cell fate through the ISR and RSR is remarkable. It is now abundantly clear that collided ribosomes, as induced by mRNA damage, serve as a “molecular sentinel” relaying signals to various pathways in response to chemical and physical stress (93, 94, 186, 276, 317, 319). Although, we have recently learned a great deal about how sensors recognize collided ribosomes, how signals cascade downstream is yet to be understood. For instance, we still do not have a clear understanding of how the collision sensor GCN1 is able to recruit and activate several factors. How the factor “chooses” to recruit GCN2 during induction of the ISR *versus* RNF25 in response to protein-mRNA crosslinks is not understood. Whether the conformations of the stalled and collided ribosomes, as well as their affinities for the various signaling factors are impacted by the type of RNA damage needs to be addressed.

Finally, it should not be forgotten that conditions that damage RNA compromise the integrity of DNA as well. Obviously, maintaining the integrity of the genome is paramount and arguably more important than that of RNA quality-control processes. Indeed, nearly all of the conditions that damage RNA discussed in this review trigger the DDR and through that cells reprogram gene expression and regulate cell fate. Interestingly, some of these outcomes overlap significantly with those activated in response to collided ribosomes. It follows then that activities of RQC, the ISR, the RSR, and DDR must somehow be coordinated. Consistent with these ideas, recent evidence suggests that in response to severe DNA damage, without contaminant RNA damage, cells can trigger cell death in a p53-indpendent manner by activating the RSR through ribosome stalling (320). Remarkably, they do so through the activation of the endonuclease SLFN11, which cleaves tRNA^Leu^_UAA_ causing stalling on UUA codons. Similarly, the ASC-1 complex, which disassembles collided ribosomes, is also involved in DNA alkylation repair (204, 226, 321). Collectively, these studies are only beginning to highlight the interplay between the various stress-signaling pathways. Understanding these networks will more than likely reveal new therapeutic targets for interventions against cancer and neurodegeneration.

## Conflicts of interest

The authors declare that they have no conflicts of interest with the contents of this article.

## References

[1] McCown P.J., Ruszkowska A., Kunkler C.N., Breger K., Hulewicz J.P., Wang M.C. (2020). Naturally occurring modified ribonucleosides. Wiley Interdiscip. Rev. RNA.

[2] Wurtmann E.J., Wolin S.L. (2009). RNA under attack: cellular handling of RNA damage. Crit. Rev. Biochem. Mol. Biol..

[3] Yan L.L., Zaher H.S. (2019). How do cells cope with RNA damage and its consequences?. J. Biol. Chem..

[4] Lindahl T. (1993). Instability and decay of the primary structure of DNA. Nature.

[5] Zaher H.S., Green R. (2009). Fidelity at the molecular level: lessons from protein synthesis. Cell.

[6] Simms C.L., Zaher H.S. (2016). Quality control of chemically damaged. RNA Cell Mol. Life Sci..

[7] Hofer T., Badouard C., Bajak E., Ravanat J.L., Mattsson A., Cotgreave I.A. (2005). Hydrogen peroxide causes greater oxidation in cellular RNA than in DNA. Biol. Chem..

[8] Yan L.L., Simms C.L., McLoughlin F., Vierstra R.D., Zaher H.S. (2019). Oxidation and alkylation stresses activate ribosome-quality control. Nat. Commun..

[9] Lindahl T. (1996). The Croonian Lecture, 1996: endogenous damage to DNA. Philos. Trans. R. Soc. Lond. B Biol. Sci..

[10] Jackson S.P., Bartek J. (2009). The DNA-damage response in human biology and disease. Nature.

[11] Ciccia A., Elledge S.J. (2010). The DNA damage response: making it safe to play with knives. Mol. Cell.

[12] Awwad S.W., Serrano-Benitez A., Thomas J.C., Gupta V., Jackson S.P. (2023). Revolutionizing DNA repair research and cancer therapy with CRISPR-Cas screens. Nat. Rev. Mol. Cell Biol..

[13] Doma M.K., Parker R. (2006). Endonucleolytic cleavage of eukaryotic mRNAs with stalls in translation elongation. Nature.

[14] Wheeler H.B., Madrigal A.A., Chaim I.A. (2024). Mapping the future of oxidative RNA damage in neurodegeneration: rethinking the status quo with new tools. Proc. Natl. Acad. Sci. U. S. A..

[15] Simms C.L., Yan L.L., Zaher H.S. (2017). Ribosome collision is critical for quality control during No-Go decay. Mol. Cell.

[16] Gilbert W.V., Bell T.A., Schaening C. (2016). Messenger RNA modifications: form, distribution, and function. Science.

[17] Gilbert W.V., Nachtergaele S. (2023). mRNA regulation by RNA modifications. Annu. Rev. Biochem..

[18] Davis F.F., Allen F.W. (1957). Ribonucleic acids from yeast which contain a fifth nucleotide. J. Biol. Chem..

[19] Wyatt G.R. (1950). Occurrence of 5-methylcytosine in nucleic acids. Nature.

[20] Sordyl D., Boileau E., Bernat A., Maiti S., Mukherjee S., Moafinejad S.N. (2025). MODOMICS: a database of RNA modifications and related information. 2025 update and 20th anniversary. Nucleic Acids Res..

[21] Ero R., Leppik M., Reier K., Liiv A., Remme J. (2024). Ribosomal RNA modification enzymes stimulate large ribosome subunit assembly in E. coli. Nucleic Acids Res..

[22] Koziej Ł., Glatt S. (2025). Mapping rRNA, tRNA, and mRNA modifications in ribosomes at high resolution. Trends Biochem. Sci..

[23] Decatur W.A., Fournier M.J. (2002). rRNA modifications and ribosome function. Trends Biochem. Sci..

[24] Sloan K.E., Warda A.S., Sharma S., Entian K.D., Lafontaine D.L.J., Bohnsack M.T. (2017). Tuning the ribosome: the influence of rRNA modification on eukaryotic ribosome biogenesis and function. RNA Biol..

[25] Alexandrov A., Chernyakov I., Gu W., Hiley S.L., Hughes T.R., Grayhack E.J. (2006). Rapid tRNA decay can result from lack of nonessential modifications. Mol. Cell.

[26] Han L., Phizicky E.M. (2018). A rationale for tRNA modification circuits in the anticodon loop. Rna.

[27] Phizicky E.M., Hopper A.K. (2010). tRNA biology charges to the front. Genes Dev..

[28] Cai Z., Song P., Yu K., Jia G. (2025). Advanced reactivity-based sequencing methods for mRNA epitranscriptome profiling. RSC Chem. Biol..

[29] Cruciani S., Novoa E.M. (2026). The new era of single-molecule RNA modification detection through nanopore base-calling models. Nat. Rev. Mol. Cell Biol..

[30] Helm M., Motorin Y. (2017). Detecting RNA modifications in the epitranscriptome: predict and validate. Nat. Rev. Genet..

[31] Wang Y., Zhang X., Liu H., Zhou X. (2021). Chemical methods and advanced sequencing technologies for deciphering mRNA modifications. Chem. Soc. Rev..

[32] Percipalle P. (2014). mRNA biogenesis, from the gene to polysomes. Semin. Cell Dev. Biol..

[33] Carrocci T.J., Neugebauer K.M. (2024). Emerging and re-emerging themes in co-transcriptional pre-mRNA splicing. Mol. Cell.

[34] Cho E.J., Takagi T., Moore C.R., Buratowski S. (1997). mRNA capping enzyme is recruited to the transcription complex by phosphorylation of the RNA polymerase II carboxy-terminal domain. Genes Dev..

[35] Jove R., Manley J.L. (1984). In vitro transcription from the adenovirus 2 major late promoter utilizing templates truncated at promoter-proximal sites. J. Biol. Chem..

[36] Rasmussen E.B., Lis J.T. (1993). In vivo transcriptional pausing and cap formation on three Drosophila heat shock genes. Proc. Natl. Acad. Sci. U S A..

[37] Ensinger M.J., Martin S.A., Paoletti E., Moss B. (1975). Modification of the 5'-terminus of mRNA by soluble guanylyl and methyl transferases from vaccinia virus. Proc. Natl. Acad. Sci. U S A..

[38] Furuichi Y., Muthukrishnan S., Tomasz J., Shatkin A.J. (1976). Mechanism of formation of reovirus mRNA 5'-terminal blocked and methylated sequence, m7GpppGmpC. J. Biol. Chem..

[39] Ramanathan A., Robb G.B., Chan S.H. (2016). mRNA capping: biological functions and applications. Nucleic Acids Res..

[40] Gonatopoulos-Pournatzis T., Cowling V.H. (2014). Cap-binding complex (CBC). Biochem. J..

[41] Pabis M., Neufeld N., Steiner M.C., Bojic T., Shav-Tal Y., Neugebauer K.M. (2013). The nuclear cap-binding complex interacts with the U4/U6·U5 tri-snRNP and promotes spliceosome assembly in mammalian cells. Rna.

[42] Flaherty S.M., Fortes P., Izaurralde E., Mattaj I.W., Gilmartin G.M. (1997). Participation of the nuclear cap binding complex in pre-mRNA 3' processing. Proc. Natl. Acad. Sci. U S A..

[43] Nojima T., Hirose T., Kimura H., Hagiwara M. (2007). The interaction between cap-binding complex and RNA export factor is required for intronless mRNA export. J. Biol. Chem..

[44] Masuda S., Das R., Cheng H., Hurt E., Dorman N., Reed R. (2005). Recruitment of the human TREX complex to mRNA during splicing. Genes Dev..

[45] Sonenberg N., Hinnebusch A.G. (2009). Regulation of translation initiation in eukaryotes: mechanisms and biological targets. Cell.

[46] Braun J.E., Truffault V., Boland A., Huntzinger E., Chang C.T., Haas G. (2012). A direct interaction between DCP1 and XRN1 couples mRNA decapping to 5' exonucleolytic degradation. Nat. Struct. Mol. Biol..

[47] Jinek M., Coyle S.M., Doudna J.A. (2011). Coupled 5' nucleotide recognition and processivity in Xrn1-mediated mRNA decay. Mol. Cell.

[48] Züst R., Cervantes-Barragan L., Habjan M., Maier R., Neuman B.W., Ziebuhr J. (2011). Ribose 2'-O-methylation provides a molecular signature for the distinction of self and non-self mRNA dependent on the RNA sensor Mda5. Nat. Immunol..

[49] Hornung V., Ellegast J., Kim S., Brzózka K., Jung A., Kato H. (2006). 5'-Triphosphate RNA is the ligand for RIG-I. Science.

[50] Akichika S., Hirano S., Shichino Y., Suzuki T., Nishimasu H., Ishitani R. (2019). Cap-specific terminal N (6)-methylation of RNA by an RNA polymerase II-associated methyltransferase. Science.

[51] Boulias K., Toczydłowska-Socha D., Hawley B.R., Liberman N., Takashima K., Zaccara S. (2019). Identification of the m(6)Am methyltransferase PCIF1 reveals the location and functions of m(6)Am in the transcriptome. Mol. Cell.

[52] Sendinc E., Valle-Garcia D., Dhall A., Chen H., Henriques T., Navarrete-Perea J. (2019). PCIF1 catalyzes m6Am mRNA methylation to regulate gene expression. Mol. Cell.

[53] Mauer J., Sindelar M., Despic V., Guez T., Hawley B.R., Vasseur J.J. (2019). FTO controls reversible m(6)Am RNA methylation during snRNA biogenesis. Nat. Chem. Biol..

[54] Mauer J., Luo X., Blanjoie A., Jiao X., Grozhik A.V., Patil D.P. (2017). Reversible methylation of m(6)A(m) in the 5' cap controls mRNA stability. Nature.

[55] Walters R.W., Matheny T., Mizoue L.S., Rao B.S., Muhlrad D., Parker R. (2017). Identification of NAD+ capped mRNAs in Saccharomyces cerevisiae. Proc. Natl. Acad. Sci. U S A..

[56] Chen Y.G., Kowtoniuk W.E., Agarwal I., Shen Y., Liu D.R. (2009). LC/MS analysis of cellular RNA reveals NAD-linked RNA. Nat. Chem. Biol..

[57] Cahová H., Winz M.L., Höfer K., Nübel G., Jäschke A. (2015). NAD captureSeq indicates NAD as a bacterial cap for a subset of regulatory RNAs. Nature.

[58] Bird J.G., Zhang Y., Tian Y., Panova N., Barvík I., Greene L. (2016). The mechanism of RNA 5′ capping with NAD+, NADH and desphospho-CoA. Nature.

[59] Bird J.G., Basu U., Kuster D., Ramachandran A., Grudzien-Nogalska E., Towheed A. (2018). Highly efficient 5' capping of mitochondrial RNA with NAD(+) and NADH by yeast and human mitochondrial RNA polymerase. Elife.

[60] Jiao X., Doamekpor S.K., Bird J.G., Nickels B.E., Tong L., Hart R.P. (2017). 5' end nicotinamide Adenine dinucleotide cap in human cells promotes RNA decay through DXO-Mediated deNADding. Cell.

[61] Cantó C., Menzies K.J., Auwerx J. (2015). NAD(+) metabolism and the control of Energy Homeostasis: a Balancing Act between Mitochondria and the Nucleus. Cell Metab..

[62] Henderson J.M., Ujita A., Hill E., Yousif-Rosales S., Smith C., Ko N. (2021). Cap 1 messenger RNA synthesis with Co-transcriptional CleanCap(®) analog by in vitro transcription. Curr. Protoc..

[63] He P.C., He C. (2021). m(6) A RNA methylation: from mechanisms to therapeutic potential. Embo J..

[64] Dominissini D., Moshitch-Moshkovitz S., Schwartz S., Salmon-Divon M., Ungar L., Osenberg S. (2012). Topology of the human and mouse m6A RNA methylomes revealed by m6A-seq. Nature.

[65] Liu J., Yue Y., Han D., Wang X., Fu Y., Zhang L. (2014). A METTL3-METTL14 complex mediates mammalian nuclear RNA N6-adenosine methylation. Nat. Chem. Biol..

[66] Ke S., Alemu E.A., Mertens C., Gantman E.C., Fak J.J., Mele A. (2015). A majority of m6A residues are in the last exons, allowing the potential for 3' UTR regulation. Genes Dev..

[67] Ping X.L., Sun B.F., Wang L., Xiao W., Yang X., Wang W.J. (2014). Mammalian WTAP is a regulatory subunit of the RNA N6-methyladenosine methyltransferase. Cell Res.

[68] Wang X., Feng J., Xue Y., Guan Z., Zhang D., Liu Z. (2016). Structural basis of N(6)-adenosine methylation by the METTL3-METTL14 complex. Nature.

[69] Wang P., Doxtader K.A., Nam Y. (2016). Structural basis for cooperative function of Mettl3 and Mettl14 methyltransferases. Mol. Cell.

[70] Desrosiers R., Friderici K., Rottman F. (1974). Identification of methylated nucleosides in messenger RNA from Novikoff hepatoma cells. Proc. Natl. Acad. Sci. U S A..

[71] Murakami S., Jaffrey S.R. (2022). Hidden codes in mRNA: control of gene expression by m(6)A. Mol. Cell.

[72] Harper J.E., Miceli S.M., Roberts R.J., Manley J.L. (1990). Sequence specificity of the human mRNA N6-adenosine methylase in vitro. Nucleic Acids Res..

[73] Kane S.E., Beemon K. (1987). Inhibition of methylation at two internal N6-methyladenosine sites caused by GAC to GAU mutations. J. Biol. Chem..

[74] Schibler U., Kelley D.E., Perry R.P. (1977). Comparison of methylated sequences in messenger RNA and heterogeneous nuclear RNA from mouse L cells. J. Mol. Biol..

[75] Wei C.M., Moss B. (1977). Nucleotide sequences at the N6-methyladenosine sites of HeLa cell messenger ribonucleic acid. Biochemistry.

[76] Jia G., Fu Y., Zhao X., Dai Q., Zheng G., Yang Y. (2011). N6-methyladenosine in nuclear RNA is a major substrate of the obesity-associated FTO. Nat. Chem. Biol..

[77] Zheng G., Dahl J.A., Niu Y., Fedorcsak P., Huang C.M., Li C.J. (2013). ALKBH5 is a mammalian RNA demethylase that impacts RNA metabolism and mouse fertility. Mol. Cell.

[78] Zhu Z.M., Huo F.C., Pei D.S. (2020). Function and evolution of RNA N6-methyladenosine modification. Int. J. Biol. Sci..

[79] Shen R., Jiang Z., Wang H., Zheng Z., Jiang X. (2025). Molecular mechanisms of m6A modifications regulating tumor radioresistance. Mol. Med..

[80] Meyer K.D., Saletore Y., Zumbo P., Elemento O., Mason C.E., Jaffrey S.R. (2012). Comprehensive analysis of mRNA methylation reveals enrichment in 3' UTRs and near stop codons. Cell.

[81] Zhou Y., Ćorović M., Hoch-Kraft P., Meiser N., Mesitov M., Körtel N. (2024). m6A sites in the coding region trigger translation-dependent mRNA decay. Mol. Cell.

[82] Kierzek E., Kierzek R. (2003). The thermodynamic stability of RNA duplexes and hairpins containing N6-alkyladenosines and 2-methylthio-N6-alkyladenosines. Nucleic Acids Res..

[83] Hudson B.H., Zaher H.S. (2015). O6-Methylguanosine leads to position-dependent effects on ribosome speed and fidelity. Rna.

[84] Choi J., Ieong K.W., Demirci H., Chen J., Petrov A., Prabhakar A. (2016). N(6)-methyladenosine in mRNA disrupts tRNA selection and translation-elongation dynamics. Nat. Struct. Mol. Biol..

[85] Ieong K.W., Indrisiunaite G., Prabhakar A., Puglisi J.D., Ehrenberg M. (2021). N 6-Methyladenosines in mRNAs reduce the accuracy of codon reading by transfer RNAs and peptide release factors. Nucleic Acids Res..

[86] Jain S., Koziej L., Poulis P., Kaczmarczyk I., Gaik M., Rawski M. (2023). Modulation of translational decoding by m(6)A modification of mRNA. Nat. Commun..

[87] Mao Y., Dong L., Liu X.M., Guo J., Ma H., Shen B. (2019). m(6)A in mRNA coding regions promotes translation via the RNA helicase-containing YTHDC2. Nat. Commun..

[88] Liu C., Sun H., Yi Y., Shen W., Li K., Xiao Y. (2023). Absolute quantification of single-base m(6)A methylation in the mammalian transcriptome using GLORI. Nat. Biotechnol..

[89] Murakami S., Olarerin-George A.O., Liu J.F., Zaccara S., Hawley B., Jaffrey S.R. (2025). m(6)A alters ribosome dynamics to initiate mRNA degradation. Cell.

[90] Ikeuchi K., Tesina P., Matsuo Y., Sugiyama T., Cheng J., Saeki Y. (2019). Collided ribosomes form a unique structural interface to induce Hel2-driven quality control pathways. Embo J..

[91] Juszkiewicz S., Chandrasekaran V., Lin Z., Kraatz S., Ramakrishnan V., Hegde R.S. (2018). ZNF598 is a quality control sensor of collided ribosomes. Mol. Cell.

[92] D'Orazio K.N., Wu C.C., Sinha N., Loll-Krippleber R., Brown G.W., Green R. (2019). The endonuclease Cue2 cleaves mRNAs at stalled ribosomes during no Go Decay. Elife.

[93] Sinha N.K., McKenney C., Yeow Z.Y., Li J.J., Nam K.H., Yaron-Barir T.M. (2024). The ribotoxic stress response drives UV-mediated cell death. Cell.

[94] Yan L.L., Zaher H.S. (2021). Ribosome quality control antagonizes the activation of the integrated stress response on colliding ribosomes. Mol. Cell.

[95] Linder B., Sharma P., Wu J., Birbaumer T., Eggers C., Murakami S. (2025). tRNA modifications tune m(6)A-dependent mRNA decay. Cell.

[96] Wang X., Zhao B.S., Roundtree I.A., Lu Z., Han D., Ma H. (2015). N(6)-methyladenosine modulates messenger RNA translation efficiency. Cell.

[97] Zaccara S., Jaffrey S.R. (2020). A unified model for the function of YTHDF proteins in regulating m(6)A-Modified mRNA. Cell.

[98] Shan T., Liu F., Wen M., Chen Z., Li S., Wang Y. (2023). m(6)A modification negatively regulates translation by switching mRNA from polysome to P-body via IGF2BP3. Mol. Cell.

[99] Meyer K.D., Patil D.P., Zhou J., Zinoviev A., Skabkin M.A., Elemento O. (2015). 5' UTR m(6)A promotes cap-independent translation. Cell.

[100] Lin S., Choe J., Du P., Triboulet R., Gregory R.I. (2016). The m(6)A methyltransferase METTL3 promotes translation in human. Cancer Cells Mol. Cell.

[101] Guca E., Alarcon R., Palo M.Z., Santos L., Alonso-Gil S., Davyt M. (2024). N(6)-methyladenosine in 5' UTR does not promote translation initiation. Mol. Cell.

[102] Bass B.L., Weintraub H. (1988). An unwinding activity that covalently modifies its double-stranded RNA substrate. Cell.

[103] Rebagliati M.R., Melton D.A. (1987). Antisense RNA injections in fertilized frog eggs reveal an RNA duplex unwinding activity. Cell.

[104] Goodman R.A., Macbeth M.R., Beal P.A. (2012). ADAR proteins: structure and catalytic mechanism. Curr. Top Microbiol. Immunol..

[105] Alseth I., Dalhus B., Bjørås M. (2014). Inosine in DNA and RNA. Curr. Opin. Genet. Dev..

[106] Gerber A.P., Keller W. (1999). An adenosine deaminase that generates inosine at the wobble position of tRNAs. Science.

[107] Pfaller C.K., George C.X., Samuel C.E. (2021). Adenosine deaminases acting on RNA (ADARs) and viral infections. Annu. Rev. Virol..

[108] Pujantell M., Riveira-Muñoz E., Badia R., Castellví M., Garcia-Vidal E., Sirera G. (2017). RNA editing by ADAR1 regulates innate and antiviral immune functions in primary macrophages. Sci. Rep..

[109] Samuel C.E. (2011). Adenosine deaminases acting on RNA (ADARs) are both antiviral and proviral. Virology.

[110] Li J.B., Walkley C.R. (2025). Leveraging genetics to understand ADAR1-mediated RNA editing in health and disease. Nat. Rev. Genet..

[111] Liddicoat B.J., Piskol R., Chalk A.M., Ramaswami G., Higuchi M., Hartner J.C. (2015). RNA editing by ADAR1 prevents MDA5 sensing of endogenous dsRNA as nonself. Science.

[112] Heraud-Farlow J.E., Chalk A.M., Linder S.E., Li Q., Taylor S., White J.M. (2017). Protein recoding by ADAR1-mediated RNA editing is not essential for normal development and homeostasis. Genome Biol..

[113] Levanon E.Y., Cohen-Fultheim R., Eisenberg E. (2024). In search of critical dsRNA targets of ADAR1. Trends Genet..

[114] Rehwinkel J., Mehdipour P. (2025). ADAR1: from basic mechanisms to inhibitors. Trends Cell Biol.

[115] Zhang Y., Bryant J., Herron L., Mali P. (2025). ADARs: pleiotropy in function, versatility in application. Nucleic Acids Res..

[116] Källman A.M., Sahlin M., Ohman M. (2003). ADAR2 A I editing: site selectivity and editing efficiency are separate events. Nucleic Acids Res..

[117] Melcher T., Maas S., Herb A., Sprengel R., Higuchi M., Seeburg P.H. (1996). RED2, a brain-specific member of the RNA-specific adenosine deaminase family. J. Biol. Chem..

[118] Jacobs M.M., Fogg R.L., Emeson R.B., Stanwood G.D. (2009). ADAR1 and ADAR2 expression and editing activity during forebrain development. Dev. Neurosci..

[119] Keegan L.P., Hajji K., O'Connell M.A. (2023). Adenosine deaminase acting on RNA (ADAR) enzymes: a journey from weird to wondrous. Acc. Chem. Res..

[120] Licht K., Hartl M., Amman F., Anrather D., Janisiw M.P., Jantsch M.F. (2019). Inosine induces context-dependent recoding and translational stalling. Nucleic Acids Res..

[121] Athanasiadis A., Rich A., Maas S. (2004). Widespread A-to-I RNA editing of alu-containing mRNAs in the human transcriptome. Plos Biol..

[122] Higuchi M., Maas S., Single F.N., Hartner J., Rozov A., Burnashev N. (2000). Point mutation in an AMPA receptor gene rescues lethality in mice deficient in the RNA-editing enzyme ADAR2. Nature.

[123] Chen M., Chen Y., Wang K., Deng X., Chen J. (2024). Non-m^6^A RNA modifications in haematological malignancies. Clin. Transl. Med..

[124] Carlile T.M., Rojas-Duran M.F., Zinshteyn B., Shin H., Bartoli K.M., Gilbert W.V. (2014). Pseudouridine profiling reveals regulated mRNA pseudouridylation in yeast and human cells. Nature.

[125] Schwartz S., Bernstein D.A., Mumbach M.R., Jovanovic M., Herbst R.H., León-Ricardo B.X. (2014). Transcriptome-wide mapping reveals widespread dynamic-regulated pseudouridylation of ncRNA and mRNA. Cell.

[126] Li X., Zhu P., Ma S., Song J., Bai J., Sun F. (2015). Chemical pulldown reveals dynamic pseudouridylation of the mammalian transcriptome. Nat. Chem. Biol..

[127] Xu H., Kong L., Cheng J., Al Moussawi K., Chen X., Iqbal A. (2024). Absolute quantitative and base-resolution sequencing reveals comprehensive landscape of pseudouridine across the human transcriptome. Nat. Methods.

[128] Maden B.E. (1990). The numerous modified nucleotides in eukaryotic ribosomal. RNA Prog. Nucleic Acid Res. Mol. Biol..

[129] Addepalli B., Limbach P.A. (2011). Mass spectrometry-based quantification of pseudouridine in RNA. J. Am. Soc. Mass Spectrom..

[130] Hamma T., Ferré-D'Amaré A.R. (2006). Pseudouridine synthases. Chem. Biol..

[131] Ofengand J., Bakin A. (1997). Mapping to nucleotide resolution of pseudouridine residues in large subunit ribosomal RNAs from representative eukaryotes, prokaryotes, archaebacteria, mitochondria and chloroplasts. J. Mol. Biol..

[132] Davis D.R. (1995). Stabilization of RNA stacking by pseudouridine. Nucleic Acids Res..

[133] Kierzek E., Malgowska M., Lisowiec J., Turner D.H., Gdaniec Z., Kierzek R. (2014). The contribution of pseudouridine to stabilities and structure of RNAs. Nucleic Acids Res..

[134] Deb I., Popenda Ł., Sarzyńska J., Małgowska M., Lahiri A., Gdaniec Z. (2019). Computational and NMR studies of RNA duplexes with an internal pseudouridine-adenosine base pair. Sci. Rep..

[135] Borchardt E.K., Martinez N.M., Gilbert W.V. (2020). Regulation and function of RNA pseudouridylation in human cells. Annu. Rev. Genet..

[136] Tomikawa C. (2025). Pseudouridine modifications in transfer RNA and tRNA pseudouridine synthases. J. Mol. Biol..

[137] Karikó K., Muramatsu H., Welsh F.A., Ludwig J., Kato H., Akira S. (2008). Incorporation of pseudouridine into mRNA yields superior nonimmunogenic vector with increased translational capacity and biological stability. Mol. Ther..

[138] Karijolich J., Yu Y.T. (2011). Converting nonsense codons into sense codons by targeted pseudouridylation. Nature.

[139] Fernández I.S., Ng C.L., Kelley A.C., Wu G., Yu Y.T., Ramakrishnan V. (2013). Unusual base pairing during the decoding of a stop codon by the ribosome. Nature.

[140] Song J., Dong L., Sun H., Luo N., Huang Q., Li K. (2023). CRISPR-free, programmable RNA pseudouridylation to suppress premature termination codons. Mol. Cell.

[141] Adachi H., Pan Y., He X., Chen J.L., Klein B., Platenburg G. (2023). Targeted pseudouridylation: an approach for suppressing nonsense mutations in disease genes. Mol. Cell.

[142] Montes M., Martínez N.M. (2023). Rewriting the message: harnessing RNA pseudouridylation to tackle disease. Mol. Cell.

[143] Eyler D.E., Franco M.K., Batool Z., Wu M.Z., Dubuke M.L., Dobosz-Bartoszek M. (2019). Pseudouridinylation of mRNA coding sequences alters translation. Proc. Natl. Acad. Sci. U S A..

[144] Kim K.Q., Burgute B.D., Tzeng S.C., Jing C., Jungers C., Zhang J. (2022). N1-methylpseudouridine found within COVID-19 mRNA vaccines produces faithful protein products. Cell Rep..

[145] Anderson B.R., Muramatsu H., Nallagatla S.R., Bevilacqua P.C., Sansing L.H., Weissman D. (2010). Incorporation of pseudouridine into mRNA enhances translation by diminishing PKR activation. Nucleic Acids Res..

[146] Wang J., Dong H., Chionh Y.H., McBee M.E., Sirirungruang S., Cunningham R.P. (2016). The role of sequence context, nucleotide pool balance and stress in 2'-deoxynucleotide misincorporation in viral, bacterial and mammalian. RNA Nucleic Acids Res..

[147] Cobley J.N., Fiorello M.L., Bailey D.M. (2018). 13 reasons why the brain is susceptible to oxidative stress. Redox Biol..

[148] Nunomura A., Perry G., Pappolla M.A., Wade R., Hirai K., Chiba S. (1999). RNA oxidation is a prominent feature of vulnerable neurons in Alzheimer's disease. J. Neurosci..

[149] Görg B., Qvartskhava N., Keitel V., Bidmon H.J., Selbach O., Schliess F. (2008). Ammonia induces RNA oxidation in cultured astrocytes and brain in vivo. Hepatology.

[150] Shan X., Chang Y., Lin C.L. (2007). Messenger RNA oxidation is an early event preceding cell death and causes reduced protein expression. Faseb J..

[151] Shan X., Tashiro H., Lin C.L. (2003). The identification and characterization of oxidized RNAs in Alzheimer's disease. J. Neurosci..

[152] Tanaka M., Chock P.B., Stadtman E.R. (2007). Oxidized messenger RNA induces translation errors. Proc. Natl. Acad. Sci. U S A..

[153] Liu M., Gong X., Alluri R.K., Wu J., Sablo T., Li Z. (2012). Characterization of RNA damage under oxidative stress in Escherichia coli. Biol. Chem..

[154] Vagbo C.B., Svaasand E.K., Aas P.A., Krokan H.E. (2013). Methylation damage to RNA induced in vivo in Escherichia coli is repaired by endogenous AlkB as part of the adaptive response. DNA Repair (Amst).

[155] Murphy M.P. (2009). How mitochondria produce reactive oxygen species. Biochem. J..

[156] Lü J.M., Lin P.H., Yao Q., Chen C. (2010). Chemical and molecular mechanisms of antioxidants: experimental approaches and model systems. J. Cell Mol. Med..

[157] Imlay J.A. (2008). Cellular defenses against superoxide and hydrogen peroxide. Annu. Rev. Biochem..

[158] Muijsers R.B., Folkerts G., Henricks P.A., Sadeghi-Hashjin G., Nijkamp F.P. (1997). Peroxynitrite: a two-faced metabolite of nitric oxide. Life Sci..

[159] Fimognari C. (2015). Role of oxidative RNA damage in chronic-degenerative. Dis. Oxid Med. Cell Longev..

[160] Dizdaroglu M., Jaruga P. (2012). Mechanisms of free radical-induced damage to DNA. Free Radic. Res..

[161] Gajewski E., Rao G., Nackerdien Z., Dizdaroglu M. (1990). Modification of DNA bases in mammalian chromatin by radiation-generated free radicals. Biochemistry.

[162] Cheng K.C., Cahill D.S., Kasai H., Nishimura S., Loeb L.A. (1992). 8-Hydroxyguanine, an abundant form of oxidative DNA damage, causes G----T and A----C substitutions. J. Biol. Chem..

[163] Nunomura A., Hofer T., Moreira P.I., Castellani R.J., Smith M.A., Perry G. (2009). RNA oxidation in Alzheimer disease and related neurodegenerative disorders. Acta Neuropathologica.

[164] Nunomura A., Tamaoki T., Motohashi N., Nakamura M., McKeel D.W., Tabaton M. (2012). The earliest stage of cognitive impairment in transition from normal aging to Alzheimer disease is marked by prominent RNA oxidation in vulnerable neurons. J. Neuropathol. Exp. Neurol..

[165] Shan X., Lin C.L. (2006). Quantification of oxidized RNAs in Alzheimer's disease. Neurobiol. Aging.

[166] Sampoli Benítez B., Barbati Z.R., Arora K., Bogdanovic J., Schlick T. (2013). How DNA polymerase X preferentially accommodates incoming dATP opposite 8-oxoguanine on the template. Biophys. J..

[167] Simms C.L., Hudson B.H., Mosior J.W., Rangwala A.S., Zaher H.S. (2014). An active role for the ribosome in determining the fate of oxidized mRNA. Cell Rep..

[168] Thomas E.N., Simms C.L., Keedy H.E., Zaher H.S. (2019). Insights into the base-pairing preferences of 8-oxoguanosine on the ribosome. Nucleic Acids Res..

[169] Rydberg B., Lindahl T. (1982). Nonenzymatic methylation of DNA by the intracellular methyl group donor S-adenosyl-L-methionine is a potentially mutagenic reaction. Embo J..

[170] García-Santos Mdel P., Calle E., Casado J. (2001). Amino acid nitrosation products as alkylating agents. J. Am. Chem. Soc..

[171] Hecht S.S. (1999). DNA adduct formation from tobacco-specific N-nitrosamines. Mutat. Res..

[172] Kohn K.W., Hartley J.A., Mattes W.B. (1987). Mechanisms of DNA sequence selective alkylation of guanine-N7 positions by nitrogen mustards. Nucleic Acids Res..

[173] Bolzán A.D., Bianchi M.S. (2002). Genotoxicity of streptozotocin. Mutat. Res..

[174] Wesolowski J.R., Rajdev P., Mukherji S.K. (2010). Temozolomide (Temodar). AJNR Am. J. Neuroradiol.

[175] Fritah S., Muller A., Jiang W., Mitra R., Sarmini M., Dieterle M. (2020). Temozolomide-Induced RNA interactome uncovers novel LncRNA regulatory loops in glioblastoma. Cancers (Basel).

[176] Wang H.T., Chen T.Y., Weng C.W., Yang C.H., Tang M.S. (2016). Acrolein preferentially damages nucleolus eliciting ribosomal stress and apoptosis in human cancer cells. Oncotarget.

[177] Drabløs F., Feyzi E., Aas P.A., Vaagbø C.B., Kavli B., Bratlie M.S. (2004). Alkylation damage in DNA and RNA--repair mechanisms and medical significance. DNA Repair (Amst).

[178] Margison G.P., Santibáñez Koref M.F., Povey A.C. (2002). Mechanisms of carcinogenicity/chemotherapy by O6-methylguanine. Mutagenesis.

[179] Koag M.C., Jung H., Kou Y., Lee S. (2019). Bypass of the major alkylative DNA lesion by human DNA Polymerase η. Molecules.

[180] Demeshkina N., Jenner L., Westhof E., Yusupov M., Yusupova G. (2012). A new understanding of the decoding principle on the ribosome. Nature.

[181] Ogle J.M., Brodersen D.E., Clemons W.M., Tarry M.J., Carter A.P., Ramakrishnan V. (2001). Recognition of cognate transfer RNA by the 30S ribosomal subunit. Science.

[182] You C., Dai X., Wang Y. (2017). Position-dependent effects of regioisomeric methylated adenine and guanine ribonucleosides on translation. Nucleic Acids Res..

[183] Thomas E.N., Kim K.Q., McHugh E.P., Marcinkiewicz T., Zaher H.S. (2020). Alkylative damage of mRNA leads to ribosome stalling and rescue by trans translation in bacteria. Elife.

[184] Small G.D., Tao M., Gordon M.P. (1968). Pyrimidine hydrates and dimers in ultraviolet-irradiated tobacco mosaic virus ribonucleic acid. J. Mol. Biol..

[185] Jackle H., Kalthoff K. (1978). Photoreactivation of RNA in UV-irradiated insect eggs (Smittia sp., Chironomidae, Diptera) I. Photosensitized production and light-dependent disappearance of pyrimidine dimers. Photochem. Photobiol..

[186] Wu C.C., Peterson A., Zinshteyn B., Regot S., Green R. (2020). Ribosome collisions trigger general stress responses to regulate cell fate. Cell.

[187] Gentile M., Latonen L., Laiho M. (2003). Cell cycle arrest and apoptosis provoked by UV radiation-induced DNA damage are transcriptionally highly divergent responses. Nucleic Acids Res..

[188] Gregersen L.H., Svejstrup J.Q. (2018). The cellular response to transcription-blocking DNA damage. Trends Biochem. Sci..

[189] Tyihák E., Albert L., Németh Z.I., Kátay G., Király-Véghely Z., Szende B. (1998). Formaldehyde cycle and the natural formaldehyde generators and capturers. Acta Biol. Hung.

[190] Liu Y., Rodriguez Y., Ross R.L., Zhao R., Watts J.A., Grunseich C. (2020). RNA abasic sites in yeast and human cells. Proc. Natl. Acad. Sci. U S A..

[191] Zhao S., Cordes J., Caban K.M., Gotz M.J., Mackens-Kiani T., Veltri A.J. (2023). RNF14-dependent atypical ubiquitylation promotes translation-coupled resolution of RNA-protein crosslinks. Mol. Cell.

[192] Mellon I., Spivak G., Hanawalt P.C. (1987). Selective removal of transcription-blocking DNA damage from the transcribed strand of the mammalian DHFR gene. Cell.

[193] Selby C.P., Sancar A. (1993). Molecular mechanism of transcription-repair coupling. Science.

[194] Fousteri M., Mullenders L.H. (2008). Transcription-coupled nucleotide excision repair in mammalian cells: molecular mechanisms and biological effects. Cell Res..

[195] Brandman O., Stewart-Ornstein J., Wong D., Larson A., Williams C.C., Li G.W. (2012). A ribosome-bound quality control complex triggers degradation of nascent peptides and signals translation stress. Cell.

[196] Simms C.L., Thomas E.N., Zaher H.S. (2017). Ribosome-based quality control of mRNA and nascent peptides. Wiley Interdiscip. Rev. RNA.

[197] Tsuboi T., Kuroha K., Kudo K., Makino S., Inoue E., Kashima I. (2012). Dom34:hbs1 plays a general role in quality-control systems by dissociation of a stalled ribosome at the 3' end of aberrant mRNA. Mol. Cell.

[198] Saito K., Horikawa W., Ito K. (2015). Inhibiting K63 polyubiquitination abolishes no-go type stalled translation surveillance in Saccharomyces cerevisiae. Plos Genet..

[199] Letzring D.P., Wolf A.S., Brule C.E., Grayhack E.J. (2013). Translation of CGA codon repeats in yeast involves quality control components and ribosomal protein L1. Rna.

[200] Winz M.L., Peil L., Turowski T.W., Rappsilber J., Tollervey D. (2019). Molecular interactions between Hel2 and RNA supporting ribosome-associated quality control. Nat. Commun..

[201] Sitron C.S., Park J.H., Brandman O. (2017). Asc1, Hel2, and Slh1 couple translation arrest to nascent chain degradation. RNA.

[202] Garzia A., Jafarnejad S.M., Meyer C., Chapat C., Gogakos T., Morozov P. (2017). The E3 ubiquitin ligase and RNA-binding protein ZNF598 orchestrates ribosome quality control of premature polyadenylated mRNAs. Nat. Commun..

[203] Juszkiewicz S., Hegde R.S. (2017). Initiation of quality control during Poly(A) translation requires site-specific ribosome ubiquitination. Mol. Cell.

[204] Sundaramoorthy E., Leonard M., Mak R., Liao J., Fulzele A., Bennett E.J. (2017). ZNF598 and RACK1 regulate Mammalian ribosome-associated quality control function by mediating regulatory 40S ribosomal ubiquitylation. Mol. Cell.

[205] Matsuo Y., Ikeuchi K., Saeki Y., Iwasaki S., Schmidt C., Udagawa T. (2017). Ubiquitination of stalled ribosome triggers ribosome-associated quality control. Nat. Commun..

[206] Buschauer R., Matsuo Y., Sugiyama T., Chen Y.H., Alhusaini N., Sweet T. (2020). The Ccr4-Not complex monitors the translating ribosome for codon optimality. Science.

[207] Frydman J., Erdjument-Bromage H., Tempst P., Hartl F.U. (1999). Co-translational domain folding as the structural basis for the rapid de novo folding of firefly luciferase. Nat. Struct. Biol..

[208] Deuerling E., Schulze-Specking A., Tomoyasu T., Mogk A., Bukau B. (1999). Trigger factor and DnaK cooperate in folding of newly synthesized proteins. Nature.

[209] Ogg S.C., Walter P. (1995). SRP samples nascent chains for the presence of signal sequences by interacting with ribosomes at a discrete step during translation elongation. Cell.

[210] Ingolia N.T., Ghaemmaghami S., Newman J.R., Weissman J.S. (2009). Genome-wide analysis in vivo of translation with nucleotide resolution using ribosome profiling. Science.

[211] Nanjaraj Urs A.N., Lasehinde V., Kim L., McDonald E., Yan L.L., Zaher H.S. (2024). Inability to rescue stalled ribosomes results in overactivation of the integrated stress response. J. Biol. Chem..

[212] Hashimoto S., Sauerland J., Zeng J., Kikuguchi C., Inada T. (2026). Parallel ZNF598 and GIGYF2 pathways mediate conserved collision-dependent mRNA decay with distinct endonucleolytic features. bioRxiv.

[213] Weber R., Chung M.Y., Keskeny C., Zinnall U., Landthaler M., Valkov E. (2020). 4EHP and GIGYF1/2 mediate translation-coupled messenger RNA decay. Cell Rep..

[214] Veltri A.J., D'Orazio K.N., Lessen L.N., Loll-Krippleber R., Brown G.W., Green R. (2022). Distinct elongation stalls during translation are linked with distinct pathways for mRNA degradation. Elife.

[215] Glover M.L., Burroughs A.M., Monem P.C., Egelhofer T.A., Pule M.N., Aravind L. (2020). NONU-1 encodes a conserved endonuclease required for mRNA translation surveillance. Cell Rep..

[216] Best K., Ikeuchi K., Kater L., Best D., Musial J., Matsuo Y. (2023). Structural basis for clearing of ribosome collisions by the RQT complex. Nat. Commun..

[217] Shoemaker C.J., Eyler D.E., Green R. (2010). Dom34:Hbs1 promotes subunit dissociation and peptidyl-tRNA drop-off to initiate no-go decay. Science.

[218] Becker T., Armache J.P., Jarasch A., Anger A.M., Villa E., Sieber H. (2011). Structure of the no-go mRNA decay complex Dom34-Hbs1 bound to a stalled 80S ribosome. Nat. Struct. Mol. Biol..

[219] Hilal T., Yamamoto H., Loerke J., Bürger J., Mielke T., Spahn C.M. (2016). Structural insights into ribosomal rescue by Dom34 and Hbs1 at near-atomic resolution. Nat. Commun..

[220] Chen L., Muhlrad D., Hauryliuk V., Cheng Z., Lim M.K., Shyp V. (2010). Structure of the Dom34-Hbs1 complex and implications for no-go decay. Nat. Struct. Mol. Biol..

[221] Guydosh N.R., Green R. (2014). Dom34 rescues ribosomes in 3' untranslated regions. Cell.

[222] Pisareva V.P., Skabkin M.A., Hellen C.U., Pestova T.V., Pisarev A.V. (2011). Dissociation by Pelota, Hbs1 and ABCE1 of mammalian vacant 80S ribosomes and stalled elongation complexes. EMBO J..

[223] Atkinson G.C., Baldauf S.L., Hauryliuk V. (2008). Evolution of nonstop, no-go and nonsense-mediated mRNA decay and their termination factor-derived components BMC. Evol. Biol..

[224] Neubauer C., Gillet R., Kelley A.C., Ramakrishnan V. (2012). Decoding in the absence of a codon by tmRNA and SmpB in the ribosome. Science.

[225] Matsuo Y., Tesina P., Nakajima S., Mizuno M., Endo A., Buschauer R. (2020). RQT complex dissociates ribosomes collided on endogenous RQC substrate SDD1. Nat. Struct. Mol. Biol..

[226] Juszkiewicz S., Speldewinde S.H., Wan L., Svejstrup J.Q., egde R.S. (2020). The ASC-1 complex disassembles collided ribosomes. Mol. Cell.

[227] Matsuo Y., Uchihashi T., Inada T. (2023). Decoding of the ubiquitin code for clearance of colliding ribosomes by the RQT complex. Nat. Commun..

[228] Stoneley M., Harvey R.F., Mulroney T.E., Mordue R., Jukes-Jones R., Cain K. (2022). Unresolved stalled ribosome complexes restrict cell-cycle progression after genotoxic stress. Mol. Cell.

[229] Yip M.C.J., Shao S. (2021). Detecting and rescuing stalled ribosomes. Trends Biochem. Sci..

[230] Bengtson M.H., Joazeiro C.A. (2010). Role of a ribosome-associated E3 ubiquitin ligase in protein quality control. Nature.

[231] Joazeiro C.A.P. (2019). Mechanisms and functions of ribosome-associated protein quality control. Nat. Rev. Mol. Cell Biol..

[232] Doamekpor S.K., Lee J.W., Hepowit N.L., Wu C., Charenton C., Leonard M. (2016). Structure and function of the yeast listerin (Ltn1) conserved N-terminal domain in binding to stalled 60S ribosomal subunits. Proc. Natl. Acad. Sci. U S A..

[233] Shen P.S., Park J., Qin Y., Li X., Parsawar K., Larson M.H. (2015). Protein synthesis. Rqc2p and 60S ribosomal subunits mediate mRNA-independent elongation of nascent chains. Science.

[234] Yonashiro R., Tahara E.B., Bengtson M.H., Khokhrina M., Lorenz H., Chen K.C. (2016). The Rqc2/Tae2 subunit of the ribosome-associated quality control (RQC) complex marks ribosome-stalled nascent polypeptide chains for aggregation. Elife.

[235] Kostova K.K., Hickey K.L., Osuna B.A., Hussmann J.A., Frost A., Weinberg D.E. (2017). CAT-tailing as a fail-safe mechanism for efficient degradation of stalled nascent polypeptides. Science.

[236] Osuna B.A., Howard C.J., Kc S., Frost A., Weinberg D.E. (2017). In vitro analysis of RQC activities provides insights into the mechanism and function of CAT tailing. Elife.

[237] Sitron C.S., Brandman O. (2019). CAT tails drive degradation of stalled polypeptides on and off the ribosome. Nat. Struct. Mol. Biol..

[238] Choe Y.J., Park S.H., Hassemer T., Körner R., Vincenz-Donnelly L., Hayer-Hartl M. (2016). Failure of RQC machinery causes protein aggregation and proteotoxic stress. Nature.

[239] Abaeva I.S., Bulakhov A.G., Hellen C.U.T., Pestova T.V. (2025). The ribosome-associated quality control factor TCF25 imposes K48 specificity on Listerin-mediated ubiquitination of nascent chains by binding and specifically orienting the acceptor ubiquitin. Genes Dev..

[240] Defenouillère Q., Yao Y., Mouaikel J., Namane A., Galopier A., Decourty L. (2013). Cdc48-associated complex bound to 60S particles is required for the clearance of aberrant translation products. Proc. Natl. Acad. Sci. U S A..

[241] Verma R., Oania R.S., Kolawa N.J., Deshaies R.J. (2013). Cdc48/p97 promotes degradation of aberrant nascent polypeptides bound to the ribosome. Elife.

[242] Verma R., Reichermeier K.M., Burroughs A.M., Oania R.S., Reitsma J.M., Aravind L. (2018). Vms1 and ANKZF1 peptidyl-tRNA hydrolases release nascent chains from stalled ribosomes. Nature.

[243] Su T., Izawa T., Thoms M., Yamashita Y., Cheng J., Berninghausen O. (2019). Structure and function of Vms1 and Arb1 in RQC and mitochondrial proteome homeostasis. Nature.

[244] Yip M.C.J., Savickas S., Gygi S.P., Shao S. (2020). ELAC1 repairs tRNAs cleaved during ribosome-associated quality control. Cell Rep..

[245] Pinto P.H., Kroupova A., Schleiffer A., Mechtler K., Jinek M., Weitzer S. (2020). ANGEL2 is a member of the CCR4 family of deadenylases with 2',3'-cyclic phosphatase activity. Science.

[246] Yip M.C.J., Keszei A.F.A., Feng Q., Chu V., McKenna M.J., Shao S. (2019). Mechanism for recycling tRNAs on stalled ribosomes. Nat. Struct. Mol. Biol..

[247] Suryo Rahmanto A., Blum C.J., Scalera C., Heidelberger J.B., Mesitov M., Horn-Ghetko D. (2023). K6-linked ubiquitylation marks formaldehyde-induced RNA-protein crosslinks for resolution. Mol. Cell.

[248] Song W., Ramadan K. (2023). Atypical K6-ubiquitin chains mobilize p97/VCP and the proteasome to resolve formaldehyde-induced RNA-protein crosslinks. Mol. Cell.

[249] Tatara Y., Kasai S., Kokubu D., Tsujita T., Mimura J., Itoh K. (2024). Emerging role of GCN1 in disease and homeostasis. Int. J. Mol. Sci..

[250] Oltion K., Carelli J.D., Yang T., See S.K., Wang H.Y., Kampmann M. (2023). An E3 ligase network engages GCN1 to promote the degradation of translation factors on stalled ribosomes. Cell.

[251] Gurzeler L.A., Link M., Ibig Y., Schmidt I., Galuba O., Schoenbett J. (2023). Drug-induced eRF1 degradation promotes readthrough and reveals a new branch of ribosome quality control. Cell Rep..

[252] Riggs C.L., Kedersha N., Ivanov P., Anderson P. (2020). Mammalian stress granules and P bodies at a glance. J. Cell Sci..

[253] Evdokimova V., Ruzanov P., Imataka H., Raught B., Svitkin Y., Ovchinnikov L.P. (2001). The major mRNA-associated protein YB-1 is a potent 5' cap-dependent mRNA stabilizer. Embo J..

[254] Budkina K., El Hage K., Clément M.J., Desforges B., Bouhss A., Joshi V. (2021). YB-1 unwinds mRNA secondary structures in vitro and negatively regulates stress granule assembly in HeLa cells. Nucleic Acids Res..

[255] Hayakawa H., Uchiumi T., Fukuda T., Ashizuka M., Kohno K., Kuwano M. (2002). Binding capacity of human YB-1 protein for RNA containing 8-oxoguanine. Biochemistry.

[256] Wu J., Jiang Z., Liu M., Gong X., Wu S., Burns C.M. (2009). Polynucleotide phosphorylase protects Escherichia coli against oxidative stress. Biochemistry.

[257] Wu J., Li Z. (2008). Human polynucleotide phosphorylase reduces oxidative RNA damage and protects HeLa cell against oxidative stress. Biochem. Biophys. Res. Commun..

[258] Ishii T., Hayakawa H., Sekiguchi T., Adachi N., Sekiguchi M. (2015). Role of Auf1 in elimination of oxidatively damaged messenger RNA in human cells. Free Radic. Biol. Med..

[259] White E.J., Matsangos A.E., Wilson G.M. (2017). AUF1 regulation of coding and noncoding. RNA Wiley Interdiscip. Rev. RNA.

[260] Ishii T., Hayakawa H., Igawa T., Sekiguchi T., Sekiguchi M. (2018). Specific binding of PCBP1 to heavily oxidized RNA to induce cell death. Proc. Natl. Acad. Sci. U S A..

[261] Zheng Q., Gan H., Yang F., Yao Y., Hao F., Hong L. (2020). Cytoplasmic m(1)A reader YTHDF3 inhibits trophoblast invasion by downregulation of m(1)A-methylated IGF1R. Cell Discov..

[262] Seo K.W., Kleiner R.E. (2020). YTHDF2 recognition of N(1)-Methyladenosine (m(1)A)-Modified RNA is associated with transcript destabilization. ACS Chem. Biol..

[263] Dai X., Wang T., Gonzalez G., Wang Y. (2018). Identification of YTH domain-containing proteins as the readers for N1-Methyladenosine. RNA Anal. Chem..

[264] Boo S.H., Ha H., Kim Y.K. (2022). m(1)A and m(6)A modifications function cooperatively to facilitate rapid mRNA degradation. Cell Rep..

[265] Xu C., Liu K., Ahmed H., Loppnau P., Schapira M., Min J. (2015). Structural basis for the discriminative recognition of N6-Methyladenosine RNA by the Human YT521-B homology domain family of proteins. J. Biol. Chem..

[266] Ma C., Liao S., Zhu Z. (2019). Crystal structure of human YTHDC2 YTH domain. Biochem. Biophys. Res. Commun..

[267] Liu H., Zeng T., He C., Rawal V.H., Zhou H., Dickinson B.C. (2022). Development of mild chemical catalysis conditions for m(1)A-to-m(6)A rearrangement on RNA. ACS Chem. Biol..

[268] Kim K.Q., Zaher H.S. (2022). Canary in a coal mine: collided ribosomes as sensors of cellular conditions. Trends Biochem. Sci..

[269] Fagard R., London I.M. (1981). Relationship between phosphorylation and activity of heme-regulated eukaryotic initiation factor 2 alpha kinase. Proc. Natl. Acad. Sci. U S A..

[270] Clemens M.J., Safer B., Merrick W.C., Anderson W.F., London I.M. (1975). Inhibition of protein synthesis in rabbit reticulocyte lysates by double-stranded RNA and oxidized glutathione: indirect mode of action on polypeptide chain initiation. Proc. Natl. Acad. Sci. U S A..

[271] Levin D.H., Petryshyn R., London I.M. (1980). Characterization of double-stranded-RNA-activated kinase that phosphorylates alpha subunit of eukaryotic initiation factor 2 (eIF-2 alpha) in reticulocyte lysates. Proc. Natl. Acad. Sci. U S A..

[272] Dever T.E., Feng L., Wek R.C., Cigan A.M., Donahue T.F., Hinnebusch A.G. (1992). Phosphorylation of initiation factor 2 alpha by protein kinase GCN2 mediates gene-specific translational control of GCN4 in yeast. Cell.

[273] Kimball S.R., Antonetti D.A., Brawley R.M., Jefferson L.S. (1991). Mechanism of inhibition of peptide chain initiation by amino acid deprivation in perfused rat liver. Regulation involving inhibition of eukaryotic initiation factor 2 alpha phosphatase activity. J. Biol. Chem..

[274] Clemens M.J., Galpine A., Austin S.A., Panniers R., Henshaw E.C., Duncan R. (1987). Regulation of polypeptide chain initiation in Chinese hamster ovary cells with a temperature-sensitive leucyl-tRNA synthetase. Changes in phosphorylation of initiation factor eIF-2 and in the activity of the guanine nucleotide exchange factor GEF. J. Biol. Chem..

[275] Hinnebusch A.G. (2005). Translational regulation of GCN4 and the general amino acid control of yeast. Annu. Rev. Microbiol..

[276] Nanjaraj Urs A.N., Kim L., Zaher H.S. (2025). Insights into the role of collided ribosomes during the activation of the integrated stress response. Biochem. Soc. Trans..

[277] Harding H.P., Zhang Y., Zeng H., Novoa I., Lu P.D., Calfon M. (2003). An integrated stress response regulates amino acid metabolism and resistance to oxidative stress. Mol. Cell.

[278] Pakos-Zebrucka K., Koryga I., Mnich K., Ljujic M., Samali A., Gorman A.M. (2016). The integrated stress response. EMBO Rep..

[279] Costa-Mattioli M., Walter P. (2020). The integrated stress response: from mechanism to disease. Science.

[280] Donnelly N., Gorman A.M., Gupta S., Samali A. (2013). The eIF2alpha kinases: their structures and functions. Cell Mol. Life Sci..

[281] Dever T.E. (2002). Gene-specific regulation by general translation factors. Cell.

[282] Loveland A.B., Bah E., Madireddy R., Zhang Y., Brilot A.F., Grigorieff N. (2016). Ribosome•RelA structures reveal the mechanism of stringent response activation. Elife.

[283] Wek R.C., Ramirez M., Jackson B.M., Hinnebusch A.G. (1990). Identification of positive-acting domains in GCN2 protein kinase required for translational activation of GCN4 expression. Mol. Cell Biol..

[284] Wek R.C., Jackson B.M., Hinnebusch A.G. (1989). Juxtaposition of domains homologous to protein kinases and histidyl-tRNA synthetases in GCN2 protein suggests a mechanism for coupling GCN4 expression to amino acid availability. Proc. Natl. Acad. Sci. U S A..

[285] Marton M.J., Vazquez de Aldana C.R., Qiu H., Chakraburtty K., Hinnebusch A.G. (1997). Evidence that GCN1 and GCN20, translational regulators of GCN4, function on elongating ribosomes in activation of eIF2alpha kinase GCN2. Mol. Cell Biol..

[286] Brown A., Fernandez I.S., Gordiyenko Y., Ramakrishnan V. (2016). Ribosome-dependent activation of stringent control. Nature.

[287] Atkinson G.C., Tenson T., Hauryliuk V. (2011). The RelA/SpoT homolog (RSH) superfamily: distribution and functional evolution of ppGpp synthetases and hydrolases across the tree of life. PLoS One.

[288] Ramirez M., Wek R.C., Vazquez de Aldana C.R., Jackson B.M., Freeman B., Hinnebusch A.G. (1992). Mutations activating the yeast eIF-2 alpha kinase GCN2: isolation of alleles altering the domain related to histidyl-tRNA synthetases. Mol. Cell Biol..

[289] Qiu H., Dong J., Hu C., Francklyn C.S., Hinnebusch A.G. (2001). The tRNA-binding moiety in GCN2 contains a dimerization domain that interacts with the kinase domain and is required for tRNA binding and kinase activation. Embo J..

[290] Dong J., Qiu H., Garcia-Barrio M., Anderson J., Hinnebusch A.G. (2000). Uncharged tRNA activates GCN2 by displacing the protein kinase moiety from a bipartite tRNA-binding domain. Mol. Cell.

[291] Lageix S., Zhang J., Rothenburg S., Hinnebusch A.G. (2015). Interaction between the tRNA-binding and C-terminal domains of Yeast Gcn2 regulates kinase activity in vivo. Plos Genet..

[292] Sattlegger E., Hinnebusch A.G. (2000). Separate domains in GCN1 for binding protein kinase GCN2 and ribosomes are required for GCN2 activation in amino acid-starved cells. Embo J..

[293] Garcia-Barrio M., Dong J., Ufano S., Hinnebusch A.G. (2000). Association of GCN1-GCN20 regulatory complex with the N-terminus of eIF2alpha kinase GCN2 is required for GCN2 activation. Embo J..

[294] Sattlegger E., Hinnebusch A.G. (2005). Polyribosome binding by GCN1 is required for full activation of eukaryotic translation initiation factor 2{alpha} kinase GCN2 during amino acid starvation. J. Biol. Chem..

[295] Yin J.Z., Keszei A.F.A., Houliston S., Filandr F., Beenstock J., Daou S. (2024). The HisRS-like domain of GCN2 is a pseudoenzyme that can bind uncharged. tRNA Struct..

[296] Han L., Guy M.P., Kon Y., Phizicky E.M. (2018). Lack of 2'-O-methylation in the tRNA anticodon loop of two phylogenetically distant yeast species activates the general amino acid control pathway. Plos Genet..

[297] Zinshteyn B., Gilbert W.V. (2013). Loss of a conserved tRNA anticodon modification perturbs cellular signaling. Plos Genet..

[298] Ishimura R., Nagy G., Dotu I., Chuang J.H., Ackerman S.L. (2016). Activation of GCN2 kinase by ribosome stalling links translation elongation with translation initiation. Elife.

[299] Harding H.P., Ordonez A., Allen F., Parts L., Inglis A.J., Williams R.L. (2019). The ribosomal P-stalk couples amino acid starvation to GCN2 activation in mammalian cells. Elife.

[300] Inglis A.J., Masson G.R., Shao S., Perisic O., McLaughlin S.H., Hegde R.S. (2019). Activation of GCN2 by the ribosomal P-stalk. Proc. Natl. Acad. Sci. U S A..

[301] Pochopien A.A., Beckert B., Kasvandik S., Berninghausen O., Beckmann R., Tenson T. (2021). Structure of Gcn1 bound to stalled and colliding 80S ribosomes. Proc. Natl. Acad. Sci. U S A..

[302] Paternoga H., Xia L., Dimitrova-Paternoga L., Li S., Yan L.L., Oestereich M. (2025). Structure of a Gcn2 dimer in complex with the large 60S ribosomal subunit. Proc. Natl. Acad. Sci. U S A..

[303] Misra J., Carlson K.R., Spandau D.F., Wek R.C. (2024). Multiple mechanisms activate GCN2 eIF2 kinase in response to diverse stress conditions. Nucleic Acids Res..

[304] Gupta R., Hinnebusch A.G. (2023). Differential requirements for P stalk components in activating yeast protein kinase Gcn2 by stalled ribosomes during stress. Proc. Natl. Acad. Sci. U S A..

[305] Kim K.Q., Li J.J., Nanjaraj Urs A.N., Pacheco M.E., Lasehinde V., Denk T. (2024). Multiprotein bridging factor 1 is required for robust activation of the integrated stress response on collided ribosomes. Mol. Cell.

[306] Zhou C., Zhang M., Murray J., Paulo J., Gygi S., Shao S. (2025). GCN1 couples GCN2 to ribosomal state to initiate amino acid response pathway signaling. Science.

[307] Meydan S., Guydosh N.R. (2020). Disome and trisome profiling reveal genome-wide targets of ribosome quality control. Mol. Cell.

[308] Harding H.P., Novoa I., Zhang Y., Zeng H., Wek R., Schapira M. (2000). Regulated translation initiation controls stress-induced gene expression in mammalian cells. Mol. Cell.

[309] Sood R., Porter A.C., Olsen D.A., Cavener D.R., Wek R.C. (2000). A mammalian homologue of GCN2 protein kinase important for translational control by phosphorylation of eukaryotic initiation factor-2alpha. Genetics.

[310] Greenberg M.E., Hermanowski A.L., Ziff E.B. (1986). Effect of protein synthesis inhibitors on growth factor activation of c-fos, c-myc, and actin gene transcription. Mol. Cell Biol..

[311] Fort P., Rech J., Vie A., Piechaczyk M., Bonnieu A., Jeanteur P. (1987). Regulation of c-fos gene expression in hamster fibroblasts: initiation and elongation of transcription and mRNA degradation. Nucleic Acids Res..

[312] Zinck R., Cahill M.A., Kracht M., Sachsenmaier C., Hipskind R.A., Nordheim A. (1995). Protein synthesis inhibitors reveal differential regulation of mitogen-activated protein kinase and stress-activated protein kinase pathways that converge on Elk-1. Mol. Cell Biol..

[313] Iordanov M.S., Pribnow D., Magun J.L., Dinh T.H., Pearson J.A., Chen S.L. (1997). Ribotoxic stress response: activation of the stress-activated protein kinase JNK1 by inhibitors of the peptidyl transferase reaction and by sequence-specific RNA damage to the alpha-sarcin/ricin loop in the 28S rRNA. Mol. Cell Biol..

[314] Iordanov M.S., Pribnow D., Magun J.L., Dinh T.H., Pearson J.A., Magun B.E. (1998). Ultraviolet radiation triggers the ribotoxic stress response in mammalian cells. J. Biol. Chem..

[315] Wang X., Mader M.M., Toth J.E., Yu X., Jin N., Campbell R.M. (2005). Complete inhibition of anisomycin and UV radiation but not cytokine induced JNK and p38 activation by an aryl-substituted dihydropyrrolopyrazole quinoline and mixed lineage kinase 7 small interfering. RNA J. Biol. Chem..

[316] Vind A.C., Snieckute G., Blasius M., Tiedje C., Krogh N., Bekker-Jensen D.B. (2020). ZAKalpha recognizes stalled ribosomes through partially redundant sensor domains. Mol. Cell.

[317] Huso V.L., Niu S., Catipovic M.A., Saba J.A., Denk T., Park E. (2025). ZAK activation at the collided ribosome. Nature.

[318] Chatterjee S., Naeli P., Onar O., Simms N., Garzia A., Hackett A. (2024). Ribosome Quality Control mitigates the cytotoxicity of ribosome collisions induced by 5-Fluorouracil. Nucleic Acids Res..

[319] Fedry J., Silva J., Vanevic M., Fronik S., Mechulam Y., Schmitt E. (2024). Visualization of translation reorganization upon persistent ribosome collision stress in mammalian cells. Mol. Cell.

[320] Boon N.J., Oliveira R.A., Korner P.R., Kochavi A., Mertens S., Malka Y. (2024). DNA damage induces p53-independent apoptosis through ribosome stalling. Science.

[321] Dango S., Mosammaparast N., Sowa M.E., Xiong L.J., Wu F., Park K. (2011). DNA unwinding by ASCC3 helicase is coupled to ALKBH3-dependent DNA alkylation repair and cancer cell proliferation. Mol. Cell.

